# Gut microbiome, cognitive function and brain structure: a multi-omics integration analysis

**DOI:** 10.1186/s40035-022-00323-z

**Published:** 2022-11-14

**Authors:** Xinxiu Liang, Yuanqing Fu, Wen-ting Cao, Zhihong Wang, Ke Zhang, Zengliang Jiang, Xiaofang Jia, Chun-ying Liu, Hong-rou Lin, Haili Zhong, Zelei Miao, Wanglong Gou, Menglei Shuai, Yujing Huang, Shengdi Chen, Bing Zhang, Yu-ming Chen, Ju-Sheng Zheng

**Affiliations:** 1 College of Life Sciences, Zhejiang University, Hangzhou, 310058 China; 2 School of Life Sciences, Westlake University, 18 Shilongshan Rd, Cloud Town, Hangzhou, 310024 China; 3 Guangdong Provincial Key Laboratory of Food, Nutrition and Health, Department of Medical Statistics and Epidemiology, School of Public Health, Sun Yat-Sen University, Guangzhou, 510080 China; 4 Chinese Center for Disease Control and Prevention, National Institute for Nutrition and Health, Beijing, 100050 China; 5 School of Public Health, Hainan Medical University, Haikou, 571199 China; 6 Westlake Intelligent Biomarker Discovery Lab, Westlake Laboratory of Life Sciences and Biomedicine, Hangzhou, 310024 China; 7 Department of Neurology and Institute of Neurology, Ruijin Hospital Affiliated to Shanghai Jiao Tong University School of Medicine, Shanghai, 200025 China; 8 Key Laboratory of Trace Element Nutrition, National Health Commission, Beijing, 100050 China; 9 Institute of Basic Medical Sciences, Westlake Institute for Advanced Study, Hangzhou, 310024 China

**Keywords:** Gut microbiome, Cognitive impairment, Brain structure, Metagenomics, Microbiome–gut–brain axis, Cohort

## Abstract

**Background:**

Microbiome-gut-brain axis may be involved in the progression of age-related cognitive impairment and relevant brain structure changes, but evidence from large human cohorts is lacking. This study was aimed to investigate the associations of gut microbiome with cognitive impairment and brain structure based on multi-omics from three independent populations.

**Methods:**

We included 1430 participants from the Guangzhou Nutrition and Health Study (GNHS) with both gut microbiome and cognitive assessment data available as a discovery cohort, of whom 272 individuals provided fecal samples twice before cognitive assessment. We selected 208 individuals with baseline microbiome data for brain magnetic resonance imaging during the follow-up visit. Fecal 16S rRNA and shotgun metagenomic sequencing, targeted serum metabolomics, and cytokine measurements were performed in the GNHS. The validation analyses were conducted in an Alzheimer’s disease case–control study (replication study 1, *n* = 90) and another community-based cohort (replication study 2, *n* = 1300) with cross-sectional dataset.

**Results:**

We found protective associations of specific gut microbial genera (*Odoribacter*, *Butyricimonas*, and *Bacteroides*) with cognitive impairment in both the discovery cohort and the replication study 1. Result of *Bacteroides* was further validated in the replication study 2. *Odoribacter* was positively associated with hippocampal volume (β, 0.16; 95% CI 0.06–0.26, *P* = 0.002), which might be mediated by acetic acids. Increased intra-individual alterations in gut microbial composition were found in participants with cognitive impairment. We also identified several serum metabolites and inflammation-associated metagenomic species and pathways linked to impaired cognition.

**Conclusions:**

Our findings reveal that specific gut microbial features are closely associated with cognitive impairment and decreased hippocampal volume, which may play an important role in dementia development.

**Supplementary Information:**

The online version contains supplementary material available at 10.1186/s40035-022-00323-z.

## Background

The number of elderly people living with dementia is rising especially in low- and middle-income countries [1]. Alzheimer’s disease (AD) is the most dominant type of dementia with cognitive impairment (also called “cognitive decline”) and brain structural alterations [1]. AD patients typically undergo several stages of cognitive impairment (preclinical, mild cognitive impairment [MCI], and dementia) before diagnosis, and the time delay between initial biochemical and cellular changes in the brain and clinical diagnosis can be more than 10 years [2]. A *Lancet* report in 2020 claimed that about 40% of dementias worldwide are related to modifiable risk factors [3]. Thus, early detection and prevention is quite important for improving the prognosis and alleviating the progression of AD, particularly for the preclinical stage or MCI [3].

Gut microbiome is essential for human health and accumulating evidence supports that gut microbial dysbiosis contributes to the pathogenesis of various neurodegenerative disorders (e.g., Parkinson’s disease and AD) [4]. Reduction of amyloid-β pathology has been observed in APP (amyloid precursor protein)/PS1 (presenilin) transgenic AD mice in the absence of intestinal microbiota, which indicates a potential role of gut microbiota in the pathogenesis of AD [5]. Several case–control studies have reported altered microbiota in both MCI and AD patients compared to normal controls [6–8]. A recent small study (*n* < 120) explored the links between microbiota profile and brain volume in people with and without obesity [9]. These findings conjointly emphasize possible effects of gut microbiome on cognitive function and brain structure, which might be further linked to the occurrence and development of dementia [10]. Nevertheless, direct evidence from large human cohorts is still lacking, leaving a research gap in this field.

Here we performed a multi-omics analysis to explore the associations of gut microbiome with age-related cognitive impairment in three independent populations. We also examined the associations of the identified gut microbial features with brain structure and volumes, as well as associations with circulating metabolites and inflammatory markers.

## Methods

### Study populations and design

### Discovery cohort

The main analyses were based on the Guangzhou Nutrition and Health Study (GNHS), a community-based prospective cohort study in southern China. Briefly, a total of 4048 individuals aged 45–72 years were enrolled during 2008–2013 and followed up every 3 years [11]. In the present study, we included 1430 participants who provided stool samples and completed cognitive screening using the Mini‐Mental State Examination (MMSE) [12] (during 2014–2019). Prior to the cognitive examination, we had collected stool samples from 272 of the included participants. During the further follow-up visit, brain images were collected via 3.0 T magnetic resonance imaging (MRI) in a subset of 208 individuals with available baseline gut microbiome data.

### Replication studies

To validate the results discovered with cognitive scores, we performed the same analysis in an AD case–control study (replication study 1; 30 AD patients, 30 MCI patients, and 30 healthy controls) which was published recently [8]. In addition, we included a total of 1300 participants with gut microbiome and cognitive assessment data (≥ 55 years) from the China Health and Nutrition Survey (CHNS) covering 15 provinces and megacities across China as replication study 2 [13].

Detailed information about the three populations was provided in the Additional file 1.

### Cognitive assessment

The MMSE, established by Folstein in 1975 [14], is one of the most widely used instruments for cognitive screening in clinical settings and epidemiologic surveys. The MMSE contains five domains, each with an assigned point value totaling 30: orientation (10 points), registration (3 points), attention and calculation (5 points), delayed recall (3 points), and language (9 points). A higher score indicates better cognitive performance [14]. In the GNHS, the participants were classified with corresponding degrees of cognitive impairment (also known as the staging model): ‘normal’ (score 30); ‘questionable’ (score 26–29); ‘mild’ (score 21–25); ‘moderate’ (score 11–20); and ‘severe’ (score 0–10) according to a validated standard [15]. As there were only 10 participants with MMSE score < 21, we assigned the participants into three groups for further statistical analyses: ‘Normal’ (score 30), ‘Questionable’ (score 26–29), and ‘Mild’ (score ≤ 25).

In the CHNS, we applied the cognitive screening items from part of the Telephone Interview for Cognitive Status–modified [16], which is a telephone adaptation of the MMSE. Cognitive performance was quantified as global cognitive score (ranging from 0 to 27 points), which was calculated as the sum of all cognitive testing items.

### Fecal microbiota DNA extraction, 16S rRNA gene sequencing, and shotgun metagenomic sequencing

In the GNHS, fecal DNA of 1430 participants was extracted according to the protocol [17]. We used the 341F (CCTACGGGNGGCWGCAG)/805R (GACTACHVGGGTATCTAATCC) primers for polymerase chain reaction (PCR) amplification of the V3–V4 regions of the 16S rRNA gene. We applied MiSeq Reagent Kits v2 (Illumina Inc., San Diego, CA) to perform amplicon sequencing on the Illuimina MiSeq System (Illumina Inc.), which generated 2 × 300 bp paired-end sequencing data with dual-index reads. Shotgun metagenomic sequencing was carried out among 1264 fecal samples from 992 individuals. Fecal samples were sequenced as one library through Illumina HiSeq machines using the 2 × 150 bp paired-end read protocol.

In the AD case–control study, fecal DNA was extracted and used for the amplification of V3–V4 regions of the 16S rRNA gene as described [8]. In the CHNS, fecal DNA extraction and 16S rRNA gene sequencing have been described in detail previously [18]. The primers 515F/806R (5′-GTGCCAGCMGCCGCGGTAA-3′/5′-GGACTACHVGGGTWTCTAAT-3′) were applied to amplify the V4 region of 16S rRNA gene.

We excluded genera (of 16S rRNA sequencing) or species (of metagenomic sequencing) that were present in < 10% of the samples or had average relative abundance < 0.01% in each dataset for further analyses. Bioinformatic analysis of gut microbiota is shown in the Additional file 1.

### Measurements of targeted serum metabolome and inflammatory cytokines in the GNHS

We performed targeted metabolomics to quantify the concentrations of 199 serum metabolites among 820 participants using an ultra-performance liquid chromatography coupled to tandem mass spectrometry system (ACQUITY UPLC-Xevo TQ-S, Waters Corp., Milford, MA).

Besides, we conducted electrochemiluminescence-based immunoassays to quantify the levels of six serum cytokines (interferon-gamma [IFN-γ], interleukin [IL]-2, IL-4, IL-6, IL-8 and IL-10) among 357 participants, using the MSD V-Plex Proinflammatory Panel 1 (human) kit (Meso Scale Diagnostics, Rockville, MD; Cat. #: K15049D-2).

### MRI acquisition, image pre-processing and voxel-based morphometry analysis in the GNHS

In the GNHS participants, 3D T1-weighted structural images were acquired with the magnetization prepared rapid acquisition gradient echo sequence on a 3.0 T scanner (MAGNETOM Skyra, Siemens Healthineers, Erlangen, Germany). We processed and analyzed 3D T1 images using MATLAB version R2020b (The MathWorks Inc, Natick, MA) and Statistical Parametric Mapping software (SPM12; The Welcome Department of Imaging Neuroscience, London). We focused on cognition-related regions of interest (ROIs) including hippocampus, superior and middle frontal lobe, and insular opercula (opercularis, orbitalis, and triangularis) [19, 20], which were identified by the Automated Anatomical Labeling atlas [21]. More information is provided in the Additional file 1.

### Statistical analysis

Statistical analysis was performed using Stata 15 (StataCorp, College Station, TX) or R software (version 4.0.4). To estimate the associations between α-diversity indices and cognitive impairment, we conducted multinomial logistic regression in the GNHS and linear mixed-effect model which contained a random intercept and random coefficient on the provinces or megacities to adjust the geographic regions in the CHNS. The covariates included age, gender, body mass index (BMI), education, and income in both the GNHS and CHNS. We performed sensitivity analysis by additionally adjusting for Bristol scale, time lag between stool sampling and cognitive assessment, and history of stroke in the GNHS. For β-diversity, we conducted principal coordinates analysis (PCoA) and permutational multivariate analysis of variance (PERMANOVA) (*vegan* function 'adonis'; 999 permutations) based on Bray–Curtis dissimilarities at the genus level in both discovery and validation cohorts.

To identify potential taxonomic biomarkers, we constructed least absolute shrinkage and selection operator (LASSO) logistic regression model (‘R’ package “glmnet”) to identify taxa that could distinguish the mild group from the normal group in the GNHS. To validate our findings, we further performed LASSO analysis between AD patients and normal controls in the case–control dataset (replication study 1). The microbes with non-zero β-coefficients achieved in both the GNHS and the AD case–control study were regarded as key genera for further validation analysis in the CNHS (replication study 2). Ratios between absolute β-coefficient of each genus and the sum of all absolute β-coefficients were calculated to quantify the contribution of each selected genus to the LASSO model. Relative abundance of genera was standardized as z-scores for the LASSO models for comparability. Considering the influence of geographic regions on gut microbiome [18], we further validated the relationships between the identified key genera and global cognitive scores using linear mixed-effect models in the CHNS, containing a random intercept and random coefficient on the provinces or megacities. In the GNHS, we then explored the association between identified key genera and cognition-related ROIs using linear mixed-effect models (False discovery rate [FDR] corrected, FDR < 0.05, adjusted for age, gender, BMI, education, income, and total intracranial volume [TIV]). Regions in the resultant T-map with FDR < 0.05 and cluster > 10 voxels were considered significant. We also extracted volumetric information of white matter (WM), grey matter (GM), cerebrospinal fluid (CSF) and each individual ROI to explore associations between key genera and the volumes using multiple linear regression adjusted for age, gender, BMI, education, income, and TIV. As short-chain fatty acids (SCFAs) might exert neuroprotective effects as prior studies reported [22], we tended to reveal latent connections between SCFAs (log-transformed) and microbiome-gut-brain axis by performing multiple linear regression analysis to investigate associations of: (1) the identified key genera with serum SCFAs adjusted for age, gender, BMI, education, and income; and (2) the SCFAs with the brain area volumes adjusted for age, gender, BMI, education, income, and TIV.

Based on the repeated measurements of gut metagenomics in the GNHS, we performed PCoA and PERMANOVA to illustrate microbial structure alterations (quantified by Bray–Curtis dissimilarities) at the species level over time across different cognitive groups. Multinomial logistic regression was used to examine the associations of intra-individual alterations in gut microbial composition with cognitive impairment. We fitted two statistical models: model 1 included age, gender, BMI, education, and income; and model 2, which was as model 1 plus Bristol scale and history of stroke.

Kruskal–Wallis test was used to compare concentrations of serum metabolite between the mild and the normal groups (or between the questionable and the normal groups). To identify metagenomic and metabolomic biomarkers that could distinguish participants with cognitive impairment from healthy controls, we constructed two LASSO models: (1) the separated models which were based on metagenomic species only, metagenomic pathways only or serum metabolites only; and (2) the combined model which was based on these three kinds of features selected in the separated models to obtain more consolidated results. We applied the semi-partial correlation [23] adjusting for age, gender, BMI, education, and income to estimate correlations between the LASSO-identified features and MMSE domains (i.e., orientation, registration, attention and calculation, delayed recall, and language); between the LASSO-identified bacterial features and the LASSO-identified serum metabolites; and between the LASSO-identified bacterial features and serum inflammatory cytokines. FDR using Benjamini–Hochberg method was calculated to correct the multiple testing.

To examine associations across multi-omics datasets, we first performed logistic regression (Mild vs. Normal) to identify significant (*P* < 0.05) genus-level features, metagenomic pathways, and serum metabolites, adjusted for age, gender, BMI, education, income, history of stroke, and time lag between stool sampling and cognitive assessment. Then we performed Spearman correlation analysis to estimate interactions among the identified multi-omics features stratified by disease state. The results were visualized in an interaction network using Gephi 0.9.2 [24]. On the other hand, we applied Spearman correlation to estimate relationships of the aforementioned significant pathways and illustrated significant (FDR < 0.05) correlations as subsets of the network.

## Results

**Fig. 1:**
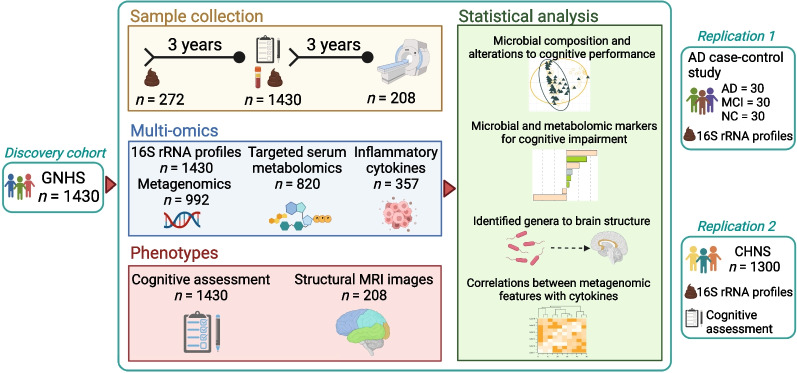
Overview of study design and analyses. We included 1430 participants from the Guangzhou Nutrition and Health Study (GNHS) as a discovery cohort, all of whom had cognitive assessment and at least one stool sample collection (272 individuals collected stool samples twice). A subset of 208 individuals underwent magnetic resonance imaging (MRI) with availability of both gut microbiome and the cognitive assessment data. Replication datasets came from the AD case–control study (*n* = 90) and the China Health and Nutrition Survey (CHNS, *n* = 1300). Image created with BioRender.com. *AD* Alzheimer’s disease, *MCI* mild cognitive impairment, *NC* normal control

The overviews of the study design and multi-omics datasets are shown in Fig. 1 and Additional file 1: Fig. S1. A total of 1430 participants from the GNHS aged 63.4 years (SD, 5.6) were enrolled in the present study, with a median MMSE score of 28 (interquartile, 26–29) (Additional file 1: Table S1). Generally, the characteristics were comparable among the subgroups stratified by cognitive function, except that the participants with lower levels of education and income were more likely to have worse cognitive performance. The mean ages were 65.2 years (SD, 6.0) in the AD case–control study [8], and 64.7 years (SD, 6.7) in the CHNS participants (Additional file 1: Table S2). In the GNHS, we finally obtained 36,149.6 paired-end reads for 16S rRNA gene sequencing and 42.4 M paired-end reads for shotgun metagenomic sequencing on average.

### Microbial features generated by 16S rRNA gene sequencing and cognitive function

**Fig. 2:**
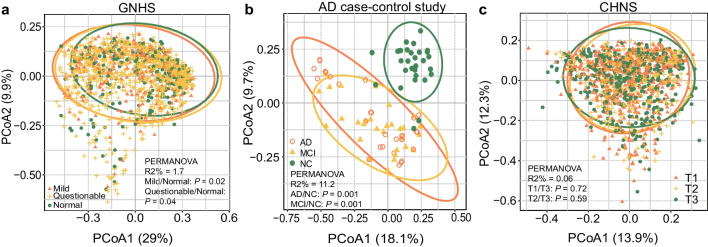
Alterations in the gut microbial structure in participants with cognitive impairment. **a**–**c** Scatterplots from principal coordinates analysis (PCoA) and permutational multivariate analysis of variance (PERMANOVA), based on Bray–Curtis distances at genus level from the GNHS, the AD case–control study and the CHNS, respectively. In the GNHS, participants were classified into corresponding degrees of cognitive impairment according to their MMSE scores: ‘Mild’ (score ≤ 25); ‘Questionable’ (score 26–29) and ‘Normal’ (score 30). *GNHS* Guangzhou Nutrition and Health Study, *AD* Alzheimer’s disease, *MCI* mild cognitive impairment, *NC* normal control, *CHNS* China Health and Nutrition Survey, *T* tertile

We identified no significant associations between α-diversity and cognitive impairment based on 16S rRNA gene sequencing data of 1430 participants in the GNHS (Additional file 1: Table S3), which were consistent with previously reported results [8]. We found a weak significance between Faith's phylogenetic diversity and global cognitive scores in the CHNS (*P* = 0.04, Additional file 1: Table S4). Distinct variances of β-diversity were observed between groups with different cognitive status in the GNHS and the AD case–control study (Fig. 2a, b), while no difference was found in the CHNS (Fig. 2c).

**Fig. 3:**
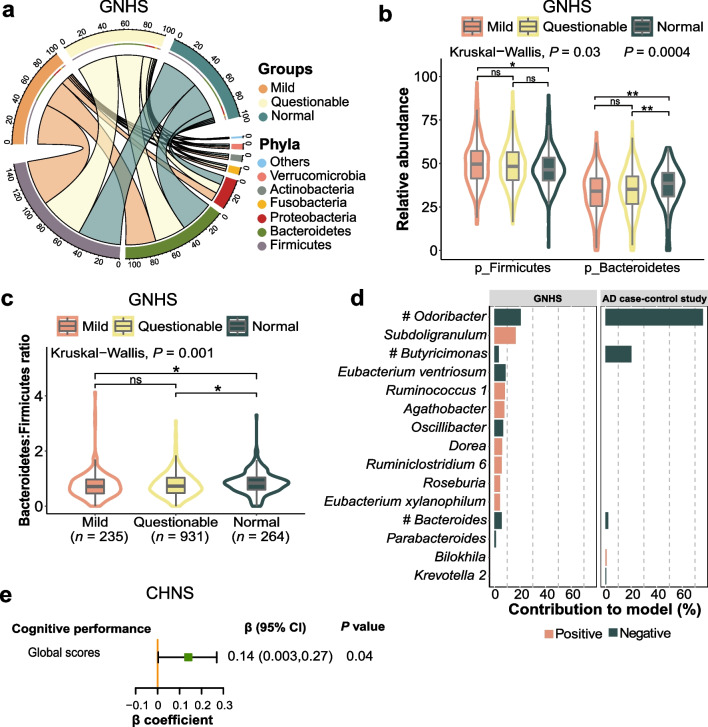
Altered phylum- and genus-level taxonomies in participants with cognitive impairment. **a** The circular layout illustrates the mean relative abundance of phyla (16S rRNA gene sequencing) among the mild, questionable, and normal groups in the GNHS. Colors in upper and lower halves of the outermost circle represent different groups of participants and microbial phyla, respectively. Width of each track highlights mean relative abundance of each phylum contained in different groups of participants. **b** and **c** The relative abundance of Firmicutes and Bacteroidetes (**b**), and the ratio of Bacteroidetes to Firmicutes (**c**) among the GNHS participants with different degrees of cognitive impairment are presented by the violin plot with included boxplot. The boxplots show median and interquartile ranges (IQR). Whiskers specify ± 1.5 × IQR. (ns, *P* > 0.05; *0.005 < *P* < 0.05; **P < 0.005; two-sided Mann–Whitney U test). **d** Key gut microbial genera that contribute to distinguishing participants with different levels of cognitive impairment (Mild *vs*. Normal in the GNHS; AD vs. NC in the AD case–control study) using LASSO models. The bars are colored according to the direction of association between the genera and cognitive impairment (orange for positive correlation [harmful]; dark green for negative correlation [beneficial]). ^**#**^Key genera selected in both populations. **e** Validation of the relationships between key genera and cognitive performance using linear mixed-effect models in the CHNS. The forest plot shows the result of the association between *Bacteroides* and global cognitive scores. The completed result is provided in the Additional file 1: Table S6. In the GNHS, participants were classified into corresponding degrees of cognitive impairment according to their MMSE scores: ‘Mild’ (score ≤ 25), ‘Questionable’ (score 26–29) and ‘Normal’ (score 30). In the CHNS, the participants were classified into T1, T2 and T3 groups according to the tertiles of their global cognitive scores. *GNHS* Guangzhou Nutrition and Health Study, *AD* Alzheimer’s disease, *LASSO* least absolute shrinkage and selection operator, *CHNS* China Health and Nutrition Survey, *T* tertile

The phylum-level compositions of gut microbiota among the mild, questionable, and normal groups were illustrated in the circular layout (Fig. 3a). The top three abundant phyla were Firmicutes, Bacteroidetes, and Proteobacteria. We found that the relative abundance of Firmicutes (*P* = 0.034) and Bacteroidetes (*P* = 0.0004), as well as the ratio of Bacteroidetes to Firmicutes (*P* = 0.001) varied significantly across the three groups. Firmicutes was more abundant in the mild group and Bacteroidetes was relatively more abundant in the normal group (Fig. 3b, c). These findings were also accordant with the previous AD case–control study [8]. Besides, the results revealed that *Odoribacter*, *Butyricimonas*, and *Bacteroides* were inversely associated with cognitive impairment in the GNHS and the AD case–control study (Fig. 3d and Additional file 1: Table S5), which were considered as key genera. Among the 3 identified key genera, we found that enrichment of *Bacteroides* was associated with better cognitive performance quantified by the global cognitive scores (β = 0.14, 95% CI 0.00–0.27) in the CHNS (Fig. 3e and Additional file 1: Table S6).

### Associations between cognition-related genera and brain structure in the GNHS

**Fig. 4:**
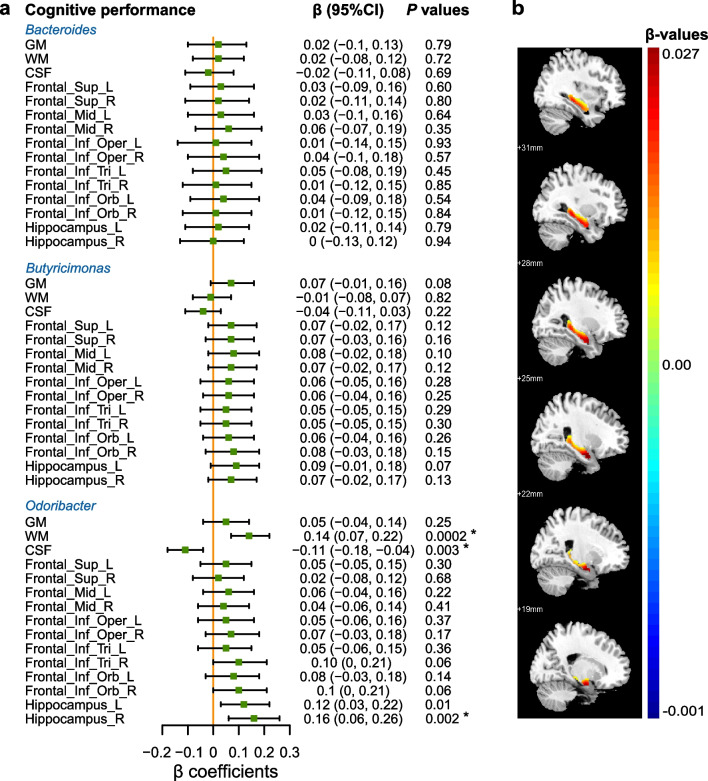
Associations between cognition-related genera and brain structure in the GNHS. **a** Linear regression was used to estimate associations between cognition-related genera and brain structure. The β-coefficients indicate the corresponding changes in standardized volumes of different brain areas for per 1-standardize unit (in SD unit) increase of the bacterial relative abundance. False discovery rate (FDR) was calculated using the Benjamini–Hochberg method. *FDR < 0.05. **b** β-Value maps of linear mixed-effect model show a positive association between the relative abundance of *Odoribacter* and the right hippocampal volume (one-way *T* test, FDR < 0.05, voxel > 10). *GNHS* Guangzhou Nutrition and Health Study, *GM* grey matter, *WM* white matter, *CSF* cerebrospinal fluid, *L* left, *R* right, *Frontal_Sup* superior frontal gyrus, dorsolateral, *Frontal_Mid* middle frontal gyrus, *Frontal_Inf_Oper* inferior frontal gyrus, opercular part, *Frontal_Inf_Tri* inferior frontal gyrus, triangular part, *Frontal_Inf_Orb* inferior frontal gyrus, orbital part

After adjustment for potential confounders, the relative abundance of *Odoribacter* was positively associated with the volumes of WM (β = 0.14, 95% CI 0.07–0.22) and the right hippocampus (β = 0.16, 95% CI 0.06–0.26), while inversely associated with CSF volume (β = − 0.11, 95% CI − 0.18 to − 0.04, Fig. 4a). Figure 4b illustrates the β coefficient between *Odoribacter* and the volume of the right hippocampus (FDR < 0.05, cluster size > 10). We also found that *Odoribacter* was positively associated with acetic acid (β = 0.07, 95% CI 0.02–0.12, Additional file 1: Table S7). The acetic acid further had a positive association with the volume of the right hippocampus (β = 0.14, 95% CI 0.01–0.27, Additional file 1: Table S8). These findings indicated a potential role of acetate in mediating the gut microbiota–hippocampus association.

### Distributions and alterations of fecal metagenome in the GNHS

**Fig. 5:**
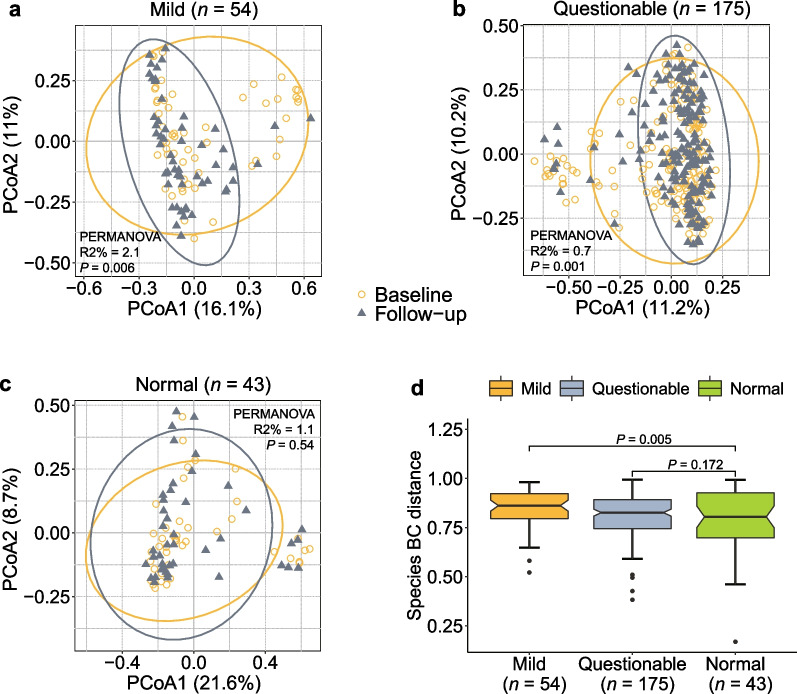
Associations between gut metagenomic alterations and cognitive function in the GNHS. **a**–**c** Principal coordinate analysis (PCoA) and permutational multivariate analysis of variance (PERMANOVA) plots of Bray–Curtis (BC) dissimilarities at the species level display the compositional alterations of gut microbiome over 3 years in groups with different cognitive status. **d** Comparison of microbial alterations quantified by Bray–Curtis dissimilarities of species among participants with different cognitive performance. *P* values were generated from multinomial logistic regression models. Boxplots show median and interquartile ranges (IQR). Whiskers specify ± 1.5 × IQR. Participants were classified into corresponding degrees of cognitive impairment according to their MMSE scores: ‘Mild’ (score ≤ 25), ‘Questionable’ (score 26–29) and ‘Normal’ (score 30). *GNHS* Guangzhou Nutrition and Health Study

A broad overview of the metagenomic taxonomy and pathways from the mild and normal groups is shown in Additional file 1: Fig. S2a (Additional file 1: Table S9). Additional file 1: Figure S2b shows significant differences in serum metabolites across the three groups (mild, questionable, and normal). Based on the repeated measurements of gut microbiome, we found that the microbial composition at the species level had a substantial alteration among individuals with cognitive impairment, but not among those with normal cognition (Fig. 5a–c). After controlling the potential covariates, we found a significantly positive association between microbial alteration (quantified by Bray–Curtis dissimilarities across the two time points) and cognitive impairment (Mild *vs.* Normal, odds ratio [OR] = 1.94, 95% CI 1.23–3.06, Additional file 1: Table S10), with the largest changes in the mild group and the smallest in the normal group (Fig. 5d). These findings demonstrated that participants with cognitive impairment may have more alterations in gut microbial composition than their normal counterparts.

### Multi-omics interactions and cognitive impairment in the GNHS

**Fig. 6:**
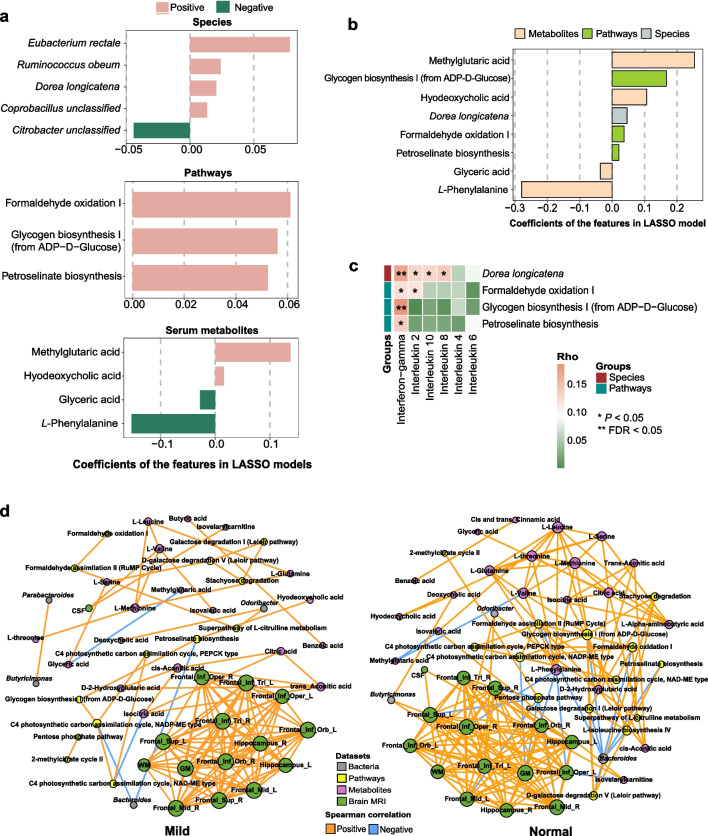
Multi-omics interactions and cognitive impairment in the GNHS. **a** and **b** Metagenomic and metabolomic markers for distinguishing participants of the mild group from the normal group using the LASSO models based on metagenomic species, pathways or serum metabolites (**a**), or the combination of these three kinds of features selected from the separated models mentioned above (**b**). The x axis denotes the coefficients of the features in each model. **c** Semi-partial correlation of key metagenomic features selected from the combined LASSO model with serum inflammatory cytokines. The intensity of color represents correlation coefficients. False discovery rate (FDR) was calculated using Benjamini–Hochberg method. **d** Significant associations among 4 aspects of multi-omics: genera of 16S rRNA gene sequencing, metagenomic pathways, serum metabolites, and brain structure. Spearman correlation was used to calculate pairwise correlations of all the measurements. Network shows significant correlations (FDR < 0.05) between each pair of measurement types. Size of nodes represents the number of connections with others. Orange edge, Spearman correlation coefficient > 0; blue edge, Spearman correlation coefficient < 0. Participants were classified into corresponding degrees of cognitive impairment according to their MMSE scores: ‘Mild’ (score ≤ 25), ‘Questionable’ (score 26–29) and ‘Normal’ (score 30). *GNHS* Guangzhou Nutrition and Health Study, *LASSO* least absolute shrinkage and selection operator, *GM* grey matter, *WM* white matter, *CSF* cerebrospinal fluid, *L* left, *R* right, *Frontal_Sup* superior frontal gyrus, dorsolateral, *Frontal_Mid* middle frontal gyrus, *Frontal_Inf_Oper* inferior frontal gyrus, opercular part, *Frontal_Inf_Tri* inferior frontal gyrus, triangular part, *Frontal_Inf_Orb* inferior frontal gyrus, orbital part

We identified 5 microbial species, 3 functional pathways, and 4 serum metabolites in LASSO models, which had potential links with age-related cognitive impairment (Fig. 6a and Additional file 1: Table S11). In the combined LASSO model, species *Dorea longicatena*, methylglutaric acid, hyodeoxycholic acid, as well as the pathways of glycogen biosynthesis I (from ADP-d-Glucose), formaldehyde oxidation I, and petroselinate biosynthesis were enriched in individuals with impaired cognition, while glyceric acid and *L*-phenylalanine were elevated in the normal controls (Fig. 6b and Additional file 1: Table S11). We further found that these cognition-related bacterial and metabolic features were significantly correlated with MMSE domain scores (Additional file 1: Fig. S3a and Additional file 1: Table S12). The species *Dorea longicatena*, the pathways of formaldehyde oxidation, and methylglutaric acid were correlated with poor scores on the MMSE domain of language. Meanwhile, hyodeoxycholic acid was negatively correlated with the scores on delayed recall domain, and glyceric acid and *L*-phenylalanine were positively associated with scores on the language domain. We found significant correlations between the identified serum metabolites (e.g., hyodeoxycholic acid and glyceric acid) and cognition-related metagenomic pathways, which may confirm readouts of the altered metagenomic pathways (Additional file 1: Fig. S3b and Additional file 1: Table S13). Moreover, these identified bacterial features also had significant correlations with serum inflammatory cytokines (Fig. 6c). Remarkably, highly abundant *Dorea longicatena* and the pathway of glycogen biosynthesis I (from ADP-d-Glucose) were correlated with elevated level of serum IFN-γ (FDR < 0.05).

After adjusting for potential confounders, we found that the pathways of formaldehyde assimilation II (RuMP Cycle) and formaldehyde oxidation I, as well as the methylglutaric acid, were enriched in samples from subjects with impaired cognition (Additional file 1: Fig. S4). Additionally, several pathways (e.g., *L*-glutamate degradation V [via hydroxyglutarate] and pentose phosphate pathway) were enriched in the normal group (Additional file 1: Fig. S4a). Individuals with normal cognition had higher levels of *L*-valine, isovelarylcarnitine, and *L*-phenylalanine (Additional file 1: Fig. S4b). As shown in Fig. 6d, the integrated network contextualizes relationships of multiple types of measurements which were associated with cognitive impairment. Both distinct and common features were found between the mild and normal groups. For example, the significant edges of the network were fewer in the mild group than in the normal group (125 vs. 186). A total of 51 and 53 nodes were included in the mild and normal groups, respectively. CSF volume was significantly associated with several metabolites including *L*-valine and isovelarylcarnitine among individuals with cognitive decline, while other brain structures (e.g., the opercular part of right inferior frontal gyrus [F rontal_Inf_Oper_R], triangular part of left inferior frontal gyrus [Frontal_Inf_Tri_L], right middle frontal gyrus [Frontal_Mid_R], and left superior frontal gyrus [Frontal_Sup_L]) were positively associated with genus *Butyricimonas* and *Odoribacter* in normal people. The degrees of amino acids had a substantial change between the mild and normal groups (16 and 60, respectively). Of note, the mild group contained six amino acids and the normal group contained eight, with six overlapping, including *L*-threonine, *L*-serine, *L*-glutamine, *L*-leucine, *L*-methionine, and *L*-valine. Additionally, the pathways of glycogen biosynthesis I (from ADP-D-Glucose) and formaldehyde oxidation I were found to play central roles in the interaction network among the normal participants, but none of the dominant pathways were found in the interaction network among the mild group. Detailed connections among cognition-related pathways are presented in Additional file 1: Fig. S5. There was a total of 15 nodes and 88 edges in the network. C4 photosynthetic carbon assimilation cycle (PEPCK type), C4 photosynthetic carbon assimilation cycle (NADP-ME type), and glycogen biosynthesis I (from ADP-D-Glucose) contributed to most of the interactions with other pathways.

## Discussion

In the present study, we found significant differences in the gut microbial composition among people with different cognitive status and revealed that increased intra-individual alterations in gut microbial composition was associated with cognitive decline. We further identified three genera including *Odoribacter*, *Butyricimonas*, and *Bacteroides* which were depleted in participants with cognitive impairment compared with normal controls. Moreover, higher abundance of *Odoribacter* was associated with several important features of brain structure, including larger volumes of WM and the right hippocampus as well as smaller CSF volume. We then revealed that chronic inflammation might underlie the associations of gut microbial features with cognitive impairment.

The associations between gut microbial α-diversity and dementia or cognitive function have been controversial. In a U.S. cohort, gut microbial α-diversity was lower in dementia patients compared with healthy controls [6], while contradictory evidence was reported in a Japanese population [25]. Notably, the sample sizes of these studies were relatively small (*n* < 130). In the present GNHS study involving more than 1000 participants, we did not find significant correlations between α-diversity and cognitive function, which was consistent with a previous study conducted in European participants [26]. Microbial instability has been associated with a variety of disease outcomes such as metabolic diseases [27] and allergenic and autoimmune disorders [28]. To the best of our knowledge, our study is the first to report that the instability of gut microbial composition is correlated with cognitive impairment. We observed significant differences in β-diversity among participants with distinct cognitive performance in two independent populations. We speculated that intra-individual alterations of microbial structure rather than α-diversity might play an important role in the cognitive maintenance.

Prior studies have demonstrated that the microbiome-gut-brain axis might be involved in the development and progression of cognitive impairment and dementia by altering permeability of the blood–brain barrier and inducing neuroinflammation [29]. SCFAs (including acetate, propionate, and butyrate) have been reported to decrease the permeability of the blood–brain barrier and exert anti-neuroinflammatory effects [22]. Of note, the three cognition-related genera identified in our study, *Odoribacter*, *Butyricimonas*, and *Bacteroides,* are all putative SCFA-producing bacteria which have potent anti-inflammatory and immunomodulatory effects [30]. Previous studies demonstrated that *Odoribacter* and *Bacteroides* are decreased in AD patients [31, 32]. *Odoribacter* has also been shown to be beneficial for hypertension prevention [33] and blood sugar regulation [34]. *Butyricimonas*, known as a protective bacterium, has the ability to produce butyric acid and isoacid salts [35]. Prior studies have reported that *Butyricimonas* is depleted in mice with spinal cord injury [36] and in patients with cystic fibrosis [37]. *Butyricimonas* is also associated with decreased adiposity and hepatic steatosis in mice [38]. Volumetric reduction of cognition-related brain areas is considered a pathological hallmark of neurodegeneration [39]. Hippocampal atrophy has been robustly linked to cognitive performance and risk of dementia [40]. In comparison to normal controls, AD patients are found with smaller white matter volume [41], while a larger volume of CSF tends to be associated with higher dementia risk [42]. Here, the associations of *Odoribacter* with the volumes of WM, right hippocampus, and CSF revealed potential neuro-protective effect of putative SCFA-producing bacteria.

The present study indicated that increased *Dorea longicatena* was associated with worse cognitive performance. *Dorea longicatena* has been reported to be positively associated with BMI and waist circumference [43]. Meanwhile, higher abundance of *Dorea longicatena* exists in individuals with circadian rhythm disturbance [44]. Recent evidence suggests that *Dorea* might contribute to elevated intestinal permeability [45]. In general, *Dorea longicatena* or *Dorea* may have a negative effect on the maintenance of a healthy gut. Furthermore, the present study revealed that *Dorea longicatena* and the pathway of formaldehyde oxidation I were positively correlated with IFN-γ. IFN-γ is an AD-related pro-inflammatory cytokine [46] and elevated IFN-γ has been reported in AD and other neurologic disorders, such as stroke and multiple sclerosis [47–49]. These results suggest that inflammation activation plays a key role in the crosstalk between gut microbiome and the central nervous system (CNS). Although IFN-γ and microglial activation have been generally linked to inflammatory stimuli in the CNS, the presence of IFN-γ in the blood is not necessarily associated with chronic inflammation [50]. Thus, our findings need to be verified by more mechanistic studies.

Besides systemic inflammation, the pathways involved in glucose metabolism and mitochondrial dysfunction have also been related with AD pathology [51, 52]. Glycogen is critical in energy and glucose metabolism [53]. Enrichment of microbial pathways of glycogen biosynthesis and degradation among participants with poor cognition in the present study may reflect the essential role of these unbalanced metabolic processes in cognitive disorders. Another pathway enriched in participants with cognitive impairment is the formaldehyde oxidation. Prior investigations have revealed that endogenous formaldehyde accumulation is mainly stimulated by aging, stroke, diabetes, and oxidative stress [54, 55]. Recent studies also support that formaldehyde exposure exhibits adverse effects on cognitive function [54]. Therefore, gut microbiota may be involved in the regulation of formaldehyde oxidation that eventually affects cognition.

In this study, we identified several key metabolites associated with cognitive impairment, of which methylglutaric acid is considered to be neurotoxic through early activation of an oxidative stress response [56] and increasing the potential for neurodegeneration in rats [57]. Another key metabolite (*L*-phenylalanine) identified is a precursor of catecholamines (including dopamine) and is essential for biosynthesis of these neurotransmitters [58]. Moreover, the concentration of *L*-phenylalanine has been found to be significantly lower in the plasma of AD patients compared to that of healthy controls [59].

There are several limitations in this study. First, the observational nature of this study makes the results subjected to the influence of potential residual confounders, and the statistically significant differences found in our analysis can only suggest associations of these multi-omics features with the outcomes. Further experimental studies or clinical trials are required to verify these findings and prove the causality. Second, this study includes only Chinese participants and thus the results may not be generalizable to other ethnic populations. Finally, although we have adjusted for the lag time (1.8 ± 1.6 years) between stool sampling and cognitive screening in the GNHS, we cannot completely rule out its potential influence on the results. A major strength of the present study is that our findings are replicated across three independent populations from different regions in China. Although the MMSE is relatively insensitive to mild/early dementia [60], we used the staging model to improve the efficiency of MMSE in identifying different stages before dementia [15]. Furthermore, the findings based on questionnaire information (i.e., MMSE) were further validated by objectively measured MRI data and successfully replicated in an independent case–control study which applied standard clinical criteria for diagnosis.

## Conclusions

Overall, the present study provides important evidence supporting the close association of gut microbiome with cognitive impairment and alterations of brain structure. The identified cognition-related gut microbial taxonomies, pathways or serum metabolites may potentially contribute to the development of interventions or drug targets for dementia and cognitive decline in the future.

## Supplementary Information

**Additional file 1: Methods.** **Table S1**. Participant characteristics in the GNHS. **Table S2**. Participant characteristics of the CHNS. **Table S3**. Associations between α-diversity and cognitive impairment in the GNHS (*n* = 1430). **Table S4**. Associations between α-diversity and cognitive decline in the CHNS (*n* = 1300). **Table S5**. Weights for genus features contributing to the LASSO models. **Table S6**. Associations between bacterial taxonomy and cognitive performance in the CHNS (*n* = 1300). **Table S7**. Associations between *Odoribacter* and SCFAs in the GNHS. **Table S8**. Associations between serum acetic acid and brain structure in the GNHS. **Table S9**. Distribution of metagenomic features in the GNHS. **Table S10**. Associations between intra-individual alterations in gut microbial composition and cognitive impairment in the GNHS. **Table S11**. Metagenomic and metabolomic features associated with cognitive impairment in the GNHS. **Table S12**. Correlations of metagenomic and metabolic features with MMSE domains in the GNHS. **Table S13**. Correlations between metagenomic features and metabolites in the GNHS. **Fig. S1** Overview of the multi-omics datasets of the GNHS. **Fig. S2** Distribution of metagenomic and metabolomic features in the GNHS. **Fig. S3** Correlation analyses on cognition-related metagenomic and metabolic traits in the GNHS. **Fig. S4** Association of metagenomic pathways and serum metabolomics with cognitive function in the GNHS. **Fig. S5** Networks of metagenomic pathways in the GNHS.

## Data Availability

The raw data for 16S rRNA gene sequences and gut metagenome of the GNHS are available in the CNSA (https://db.cngb.org/cnsa/) of CNGBdb at accession number CNP0000829 and CNP0001510, respectively. Data of the Alzheimer’ disease case–control study are availabe in the National Center for Biotechnology Information (NCBI) BioProject database with project number PRJNA489760. Other data described in the article will be made available upon request by bona fide researchers for specified scientific purposes via contacting the corresponding authors.

## Abbreviations

AD: Alzheimer’s disease
MCI: Mild cognitive impairment
GNHS: The Guangzhou Nutrition and Health Study
MMSE: Mini-mental state examination
MRI: Magnetic resonance imaging
CHNS: The China Health and Nutrition Survey
IFN-γ: Interferon-gamma
IL: Interleukin
ROI: Region of interest
Q: Quartile
BMI: Body mass index
PCoA: Principal coordinates analysis
BC: Bray–Curtis
PERMANOVA: Permutational multivariate analysis of variance
LASSO: Least absolute shrinkage and selection operator
FDR: False discovery rate
TIV: Total intracranial volume
WM: White matter
GM: Grey matter
CSF: Cerebrospinal fluid
SCFA: Short-chain fatty acid
OR: Odds ratio
Frontal_Sup: Superior frontal gyrus: dorsolateral
Frontal_Mid: Middle frontal gyrus
Frontal_Inf_Oper: Inferior frontal gyrus: opercular part
Frontal_Inf_Tri: Inferior frontal gyrus: triangular part
Frontal_Inf_Orb: Inferior frontal gyrus: orbital part
CNS: Central nervous system
