# Supplementary material for: Gut microbiome, cognitive function and brain structure: a multi-omics integration analysis
Source: Transl Neurodegener. 2022 Nov 14;11:49. doi: 10.1186/s40035-022-00323-z (PMC9661756; doi:10.1186/s40035-022-00323-z)
Supplement: Supplementary file 1 — Additional file 1: Methods. Table S1. Participant characteristics in the GNHS. Table S2. Participant characteristics of the CHNS. Table S3. Associations between α-diversity and cognitive impairment in the GNHS (n = 1430). Table S4. Associations between α-diversity and cognitive decline in the CHNS (n = 1300). Table S5. Weights for genus features contributing to the LASSO models. Table S6. Associations between bacterial taxonomy and cognitive performance in the CHNS (n = 1300). Table S7. Associations between Odoribacter and SCFAs in the GNHS. Table S8. Associations between serum acetic acid and brain structure in the GNHS. Table S9. Distribution of metagenomic features in the GNHS. Table S10. Associations between intra-individual alterations in gut microbial composition and cognitive impairment in the GNHS. Table S11. Metagenomic and metabolomic features associated with cognitive impairment in the GNHS. Table S12. Correlations of metagenomic and metabolic features with MMSE domains in the GNHS. Table S13. Correlations between metagenomic features and metabolites in the GNHS. Fig. S1 Overview of the multi-omics datasets of the GNHS. Fig. S2 Distribution of metagenomic and metabolomic features in the GNHS. Fig. S3 Correlation analyses on cognition-related metagenomic and metabolic traits in the GNHS. Fig. S4 Association of metagenomic pathways and serum metabolomics with cognitive function in the GNHS. Fig. S5 Networks of metagenomic pathways in the GNHS. [file 40035_2022_323_MOESM1_ESM.docx]

**Supplementary Material for:**

**“*Gut microbiome, cognitive function and brain structure: a multi-omics integration analysis*”**

**Supplementary Methods**

**Study** **population and design**

Discovery cohort

In the Guangzhou Nutrition and Health Study (GNHS), we screened 1486 participants who provided stool samples and completed cognitive screening using the Mini‐Mental State Examination (MMSE)(1). There was a median lag time of 1.8 years (SD: 1.6) between stool sampling and cognitive screening. With a 3-year interval, a subset of 278 individuals had stool samples collected twice before the cognitive examination. We excluded individuals with self-reported cancers (*n* = 31), Parkinson’s disease (*n* = 6), antibiotic use within 2 weeks of stool sampling (*n* = 13), or missing information of key covariates, including age, gender, body mass index (BMI), education, income or history of stroke (*n* = 6). Finally, 1430 participants were included in the present analysis, of whom 272 individuals had repeated collection of stool samples before cognitive examination. The venous whole blood samples were collected after overnight fasting using serum separation tubes, and then centrifuged at 3,500 rpm for 10 minutes for serum collection. Serum aliquots were stored at –80°C for further detection. The stool samples were collected at a local study site within the School of Public Health at Sun Yat-sen University during follow-up visits of the GNHS, and transferred to a –80°C freezer within 4 hours after collection.

Replication studies

We included data from two independent studies to replicate the findings from the GNHS. To validate the results discovered with cognitive scores only, we performed the same analysis in an AD case-control study (30 AD patients, 30 MCI patients, and 30 healthy controls) which was published recently.(2) The AD case-control study obtained host metadata and fecal 16S rRNA gene sequencing. This study was conducted among Chinese participants in Shanghai, eastern China, and the detailed information has been described previously(2).

To further confirm our findings, we included data from an independent cohort–the China Health and Nutrition Survey (CHNS) as a further replication dataset. CHNS is a household-based prospective cohort study established in 1989, which recruited participants across 12 provinces and 3 megacities of China(3). The cohort was designed to longitudinally collect information about demographics, socioeconomics, lifestyle, physical activity, diet, medication and health. We first included 1358 participants with gut microbiota profiles and cognitive assessment completed in 2015 (≥ 55 years). We excluded individuals with antibiotic use within 2 weeks of stool sampling (*n* = 58). Finally, 1300 people were included in the present analysis with 26 and 5 people missing data on household income and BMI, respectively. We used the modal numbers to replace missing values of income, and the mean values calculated across the remaining participants to replace missing values of BMI. The cognitive screening items were consisted of part of the Telephone Interview for Cognitive Status–modified(4), which mainly evaluated immediate and delayed recall of a 10-word list (scores 0–10 each), attention and calculation ability by examining counting backward from 20, and serial 7 subtractions (scores 1–7). Higher scores for all items indicated better cognitive performance. The cognitive performance was quantified as repeated measures of the global cognitive score (ranging from 0 to 27 points) which was calculated as the sum of the scores of all cognitive testing items. Each participant received adequate training and instructions about the collection process before collecting stool samples. Stool samples were frozen at –20°C temporally and transported by a cold chain to the central laboratory within 24–48 hours. Then all stool samples were stored in a −20°C facility.

**Bioinformatic analysis of 16S rRNA gene sequencing and shotgun metagenomic sequencing**

We used QIIME2 2019.04 for merging paired reads, filtering reads of low quality and human genomic DNA, and analyzing sequences both in the GNHS and CHNS(5). α-diversity was evaluated with sampling depth of 1000. We trained Naïve–Bayes classifier on the SILVA v132 database at 99% similarity for taxonomic assignment. Genera with prevalence less than 10% of samples and average relative abundance less than 0.01% were excluded. Ultimately, 88 and 146 genera were included for further analysis in the GNHS and CHNS, respectively.

In the GNHS, 1264 fecal samples from 992 participants were sequenced (shotgun metagenome) as one library through Illumina HiSeq machines using the 2×150 bp paired-end read protocol. We applied PRINSEQ v0.20.4 for data quality control. Reads aligned to the human reference genome (H. sapiens, UCSC hg19) were eliminated (aligned with Bowtie2 v2.2.5). We carried out MetaPhlAn2 v2.6.02(6) with default settings for the metagenomic taxonomy analysis. Functional profiling was performed with HUMAnN2 v2.8.1 which reconstructed metabolic pathway abundance according to the subset of metabolic reaction-related gene families from MetaCyc(7). Detailed bioinformatic analysis of metagenomics was described previously(8). We annotated a total of 11 phyla, 220 genera, 549 species and 509 pathways. We restricted the analyses to 157 species with a prevalence of 10% or higher and average relative abundance of more than 0.01%, and 368 pathways with prevalence 10% or higher.

**MRI acquisition, image pre-processing and voxel-based morphometry (VBM) analysis in the GNHS**

In the GNHS participants, 3D T1-weighted structural images were acquired with the magnetization prepared rapid acquisition gradient echo (MPRAGE) sequence on a 3.0T scanner (MAGNETOM Skyra, Siemens Healthineers, Erlangen, Germany): echo time (TE) = 2.19 ms, repetition time (TR) = 2300 ms, flip angle = 8°, field of view (FOV) 256×256 mm, acquisition matrix = 256×256, thickness 1.0 mm, 176 slices,(9) and 1.00 mm cubic voxel size. Participants were required to open eyes and keep awake while scanning. We processed and analyzed the 3D T1 images using MATLAB version R2020b (The MathWorks Inc, Natick, Mass) and Statistical Parametric Mapping software (SPM12; The Welcome Department of Imaging Neuroscience, London).

SPM12 was applied to perform segmentation, registration, normalization, and spatial smoothing. Images for each participant were segmented into grey matter (GM), white matter (WM), and cerebrospinal fluid (CSF). Diffeomorphic Anatomical Registration Through Exponentiated Lie Algebra (DARTEL) algorithm was applied to normalize GM into Montreal Neurological Institute (MNI) space using default brain template (resampling voxel size = 1.5 mm×1.5 mm×1.5 mm). Individual flow fields were used to warp the corresponding GM segment. As a final step, the resulting images were modulated and smoothed using an 8-mm full-width at half maximum (FWHM) isotropic Gaussian kernel. We focused on cognition-related regions of interest (ROIs) including hippocampus, superior and middle frontal lobe, and insular opercula (opercularis, orbitalis, and triangularis),(9, 10) which were identified by the Automated Anatomical Labeling (AAL) atlas.(11)

**Table S1. Participant characteristics** **in the GNHS**

| **Characteristics** | **Total** | **Degrees of cognitive impairment**^*^ | | | ***P* values**^†^ |
| --- | --- | --- | --- | --- | --- |
|  |  | **Mild** | **Questionable** | **Normal** |  |
| No. of participants | 1430 | 235 | 931 | 264 |  |
| Age (years), mean (SD) | 63.4 (5.6) | 63.5 (5.8) | 63.5 (5.7) | 62.8 (5.1) | 0.13 |
| Women, n (%) | 944 (66.0) | 155 (66.0) | 609 (65.4) | 180 (68.2) | 0.70 |
| BMI (kg/m^2^), mean (SD) | 23.5 (3.3) | 23.3 (3.3) | 23.6 (3.4) | 23.5 (3.4) | 0.68 |
| Education, n (%) |  |  |  |  | <0.01 |
| < High school | 354 (24.8) | 80 (34.0) | 230 (24.7) | 44 (16.7) |  |
| High school or vocational school | 676 (47.3) | 111 (47.2) | 439 (47.2) | 126 (47.7) |  |
| College or professional school | 400 (28.0) | 44 (18.7) | 262 (28.1) | 94 (35.6) |  |
| Income, n (%) |  |  |  |  | <0.01 |
| <501 ¥/month | 23 (1.6) | 5 (2.1) | 15 (1.6) | 3 (1.1) |  |
| 501–1500 ¥/month | 335 (23.4) | 72 (30.6) | 205 (22.0) | 58 (22.0) |  |
| 1501–3000 ¥/month | 867 (60.6) | 135 (57.4) | 581 (62.4) | 151 (57.2) |  |
| >3000 ¥/month | 205 (14.3) | 23 (9.8) | 130 (14.0) | 52 (19.7) |  |
| Current smoking, n (%) | 107 (7.5) | 22 (9.4) | 72 (7.7) | 13 (4.9) | 0.15 |
| Current alcohol drinking, n (%) | 109 (7.6) | 14 (6.0) | 72 (7.7) | 23 (8.7) | 0.50 |
| Physical activity (METs), mean (SD) ^‡^ | 41.1 (14.6) | 41.0 (15.2) | 41.0 (14.3) | 41.1 (14.6) | 0.08 |
| Energy intake (kcal/d), mean (SD) ^‡^ | 1802.8 (570.3) | 1762.7 (652.4) | 1807.6 (560.1) | 1821.7 (526.9) | 0.47 |
| Type 2 diabetes, n (%) | 202 (14.1) | 33 (14.0) | 132 (14.2) | 37 (14.0) | 0.99 |
| Hypertension, n (%) | 519 (36.3) | 102 (43.4) | 326 (35.0) | 91 (34.5) | 0.05 |
| Stroke, n (%) | 47 (3.3) | 16 (6.8) | 24 (2.6) | 7 (2.7) | <0.01 |
| MMSE, median (IQR) | 28 (26–29) | 24 (23–25) | 28 (27–29) | 30 (30–30) | <0.01 |
| MMSE subscores, median (IQR) |  |  |  |  |  |
| Orientation | 10 (10–10) | 10 (10–10) | 10 (10–10) | 10 (10–10) | <0.01 |
| Registration | 3 (3–3) | 3 (3–3) | 3 (3–3) | 3 (3–3) | <0.01 |
| Attention and calculation | 5 (4–5) | 2 (1–3) | 5 (4–5) | 5 (5–5) | <0.01 |
| Recall | 2 (2–3) | 1 (1–2) | 2 (2–3) | 3 (3–3) | <0.01 |
| Language | 9 (8–9) | 7 (7–8) | 9 (8–9) | 9 (9–9) | <0.01 |

Abbreviations: GNHS, Guangzhou Nutrition and Health Study; BMI, body mass index; MET, metabolic equivalent; SD, standard deviation; MMSE, Mini-Mental State Examination; IQR, interquartile range.

^*^ Participants were classified into corresponding degrees of cognitive impairment according to their MMSE scores: ‘Mild’ (score ≤25); ‘Questionable’ (score 26–29) and ‘Normal’ (score 30).

^†^ *P* values were generated from Pearson's chi-squared for categorical variables, analysis of variance (ANOVA) for normal quantitative, and Kruskal–Wallis test for non-normal quantitative variables.

^‡^ The information of physical activity and energy intake was collected in 2008 (baseline of the GNHS).

**Table S2. Participant characteristics of the CHNS**

| **Characteristics** | **Total** | **Global cognitive scores** | | |  |
| --- | --- | --- | --- | --- | --- |
|  |  | **T1** | **T2** | **T3** | ***P* values^*^** |
| No. of participants | 1300 | 494 | 371 | 435 |  |
| Age (years), mean (SD) | 63.7 (6.0) | 64.7 (6.7) | 63.2 (5.4) | 62.9 (5.5) | <0.01 |
| Women, n (%) | 666 (51.2%) | 298 (60.3%) | 173 (46.6%) | 195 (44.8%) | <0.01 |
| BMI (kg/m^2^), mean (SD) | 24.2 (4.2) | 24.2 (5.1) | 24.2 (3.7) | 24.3 (3.3) | 0.86 |
| Education, n (%) |  |  |  |  | <0.01 |
| < High school | 966 (74.3%) | 414 (83.8%) | 269 (72.5%) | 283 (65.1%) |  |
| High school or vocational school | 264 (20.3%) | 68 (13.8%) | 79 (21.3%) | 117 (26.9%) |  |
| College or professional school | 70 (5.4%) | 12 (2.4%) | 23 (6.2%) | 35 (8.0%) |  |
| Income, ¥/year | 61445 (78127) | 55260 (80611) | 61733 (81048) | 68105 (72188) | 0.05 |
| Current smoking, n (%) | 301 (23.2%) | 98 (19.8%) | 87 (23.5%) | 116 (26.7%) | 0.05 |
| Current alcohol drinking, n (%) | 290 (22.3%) | 102 (20.6%) | 92 (24.8%) | 96 (22.1%) | 0.35 |
| Physical activity, n (%) |  |  |  |  | 0.83 |
| Low | 434 (33.4%) | 172 (34.8%) | 117 (31.5%) | 145 (33.3%) |  |
| Medium | 433 (33.3%) | 161 (32.6%) | 123 (33.2%) | 149 (34.3%) |  |
| High | 433 (33.3%) | 161 (32.6%) | 131 (35.3%) | 141 (32.4%) |  |
| Energy intake (kcal/d), mean (SD) | 1938.3 (755.9) | 1883.0 (890.7) | 1919.0 (612.4) | 2017.4 (692.2) | 0.02 |
| Hypertension, n (%) | 310 (23.8%) | 116 (23.5%) | 87 (23.5%) | 107 (24.6%) | 0.90 |
| Type 2 diabetes, n (%) | 234 (18.0%) | 90 (18.2%) | 64 (17.3%) | 80 (18.4%) | 0.90 |

Abbreviations: CHNS, China Health and Nutrition Survey; T, tertile; BMI, body mass index; SD, standard deviation.

^*^ *P* values were generated from Pearson's chi-squared for categorical variables, analysis of variance (ANOVA) for normal quantitative, and Kruskal–Wallis test for non-normal quantitative variables.

**Table S3.** **Associations between α-diversity and cognitive impairment in the GNHS (*n* = 1430)**

| **α-diversity** | **Questionable *vs.* Normal** | |  | **Mild *vs.* Normal** | |
| --- | --- | --- | --- | --- | --- |
|  | **OR (95% CI)** ^*^ | ***P* values** |  | **OR (95% CI)** ^*^ | ***P* values** |
| Shannon diversity index |  |  |  |  |  |
| Model 1^†^ | 0.96 (0.76, 1.21) | 0.74 |  | 1.00 (0.75, 1.35) | 0.98 |
| Model 2^†^ | 0.93 (0.74, 1.18) | 0.56 |  | 0.94 (0.69, 1.28) | 0.70 |
| Faith's PD |  |  |  |  |  |
| Model 1 | 0.98 (0.94, 1.03) | 0.43 |  | 1.01 (0.95, 1.07) | 0.85 |
| Model 2 | 0.98 (0.93, 1.02) | 0.34 |  | 0.99 (0.93, 1.06) | 0.86 |
| Observed OTUs |  |  |  |  |  |
| Model 1 | 1.00 (0.99, 1.01) | 0.98 |  | 1.00 (0.99, 1.01) | 0.70 |
| Model 2 | 1.00 (0.99, 1.01) | 0.98 |  | 1.00 (0.99, 1.01) | 0.82 |
| Pielou's evenness |  |  |  |  |  |
| Model 1 | 0.63 (0.06, 7.11) | 0.71 |  | 0.74 (0.03, 16.72) | 0.85 |
| Model 2 | 0.40 (0.03, 4.77) | 0.47 |  | 0.33 (0.01, 8.12) | 0.50 |

Abbreviations: GNHS, Guangzhou Nutrition and Health Study; OR, odds ratio; CI, confidence interval; PD, phylogenetic diversity; OTU, operational taxonomic units.

^*^ ORs were generated from multinomial logistic regression with the normal group as the reference.

^†^ The model 1 was adjusted for age, gender, body mass index, education, and income; and the model 2, as model 1 + Bristol scale, time lag between stool sampling and cognitive assessment, and history of stroke.

**Table S4. Associations between α-diversity and cognitive decline in the CHNS (*n* = 1300)**

| **α-diversity** |  | **β (95% CI)**^*^ | ***P* values** |
| --- | --- | --- | --- |
| Shannon diversity index |  | 0.31 (-0.15, 0.76) | 0.19 |
| Faith's PD |  | 0.13 (0.01, 0.25) | 0.04 |
| Observed OTUs |  | 0.01 (0.00, 0.02) | 0.13 |
| Pielou's evenness |  | 2.55 (-1.69, 6.79) | 0.24 |

Abbreviations: CHNS, China Health and Nutrition Survey; T, tertile; OR, odds ratio; CI, confidence interval; PD, phylogenetic diversity; OTU, operational taxonomic units.

^*^ β-coefficients were generated from linear mixed-effect models.

^†^ The covariates included age, gender, body mass index, education, and income.

**Table S5. Weights for genus features contributing to the LASSO models**

| **Genera^*^** | **β coefficients** | **Contribution to model (%)** |
| --- | --- | --- |
| **GNHS (Mild *vs.* Normal)^†^** |  |  |
| *Eubacterium ventriosum* | -0.040 | 8.801 |
| *Oscillibacter* | -0.032 | 6.915 |
| *Bacteroides* | -0.027 | 5.796 |
| *Butyricimonas* | -0.016 | 3.447 |
| *Odoribacter* | -0.094 | 20.588 |
| *Parabacteroides* | -0.005 | 1.186 |
| *Eubacterium xylanophilum* | 0.020 | 4.286 |
| *Roseburia* | 0.021 | 4.483 |
| *Ruminiclostridium 6* | 0.026 | 5.758 |
| *Dorea* | 0.028 | 6.083 |
| *Agathobacter* | 0.035 | 7.737 |
| *Ruminococcus 1* | 0.038 | 8.300 |
| *Subdoligranulum* | 0.076 | 16.620 |
| **AD case-control study (AD *vs.* NC)^†^** |  |  |
| *Bacteroides* | -0.040 | 2.071 |
| *Butyricimonas* | -0.394 | 20.289 |
| *Odoribacter* | -1.481 | 76.152 |
| *Krevotella 2* | -0.012 | 0.600 |
| *Bilokhila* | 0.017 | 0.888 |

Abbreviations: LASSO, least absolute shrinkage and selection operator; GNHS, Guangzhou Nutrition and Health Study; AD, Alzheimer's disease; MCI, mild cognitive impairment; NC, normal control.

^*^ Z-scores of relative abundance of the genera were calculated before performing LASSO analysis.

^†^ The degrees of cognitive impairment in the GNHS cohort were quantified according to MMSE scores: 'Mild' (score ≤ 25), 'Questionable' (score 26–29) and 'Normal' (score 30). In the AD case-control study, the AD and MCI were diagnosed according to the criteria of the National Institute on Aging-Alzheimer’s Association (NIA-AA) workgroups, and the NC was cognitively normal controls.

**Table S6. Associations between bacterial taxonomy and cognitive performance in the CHNS (*n* = 1300)**

| **Genera** |  | **β (95% CI)** ^*^ | ***P* values** |
| --- | --- | --- | --- |
| ***Bacteroides*** |  | 0.14 (0.00, 0.27) | 0.04 |
| ***Butyricimonas*** |  | -3.19 (-24.46, 18.08) | 0.77 |
| ***Odoribacter*** |  | 10.67 (-8.69, 30.02) | 0.28 |

Abbreviations: CHNS, China Health and Nutrition Survey; T, tertile; OR, odds ratio; CI, confidence interval.

^*^ β-coefficients were generated linear mixed-effect models with the global cognitive score as the outcome.

^†^ The covariates included age, gender, body mass index, education, and income.

**Table S7. Associations between *Odoribacter* and SCFAs in the GNHS**

| **SCFAs** | **No. of participants** | **β (95% CI)^*^** | ***P* values** |
| --- | --- | --- | --- |
| Acetic acid |  |  |  |
| Model 1^†^ | 820 | 0.07 (0.02, 0.12) | 0.01 |
| Model 2^†^ | 820 | 0.07 (0.02, 0.12) | 0.01 |
| Propanoic acid |  |  |  |
| Model 1 | 820 | 0.01 (-0.01, 0.03) | 0.46 |
| Model 2 | 820 | 0.01 (-0.01, 0.03) | 0.51 |
| Butyric acid |  |  |  |
| Model 1 | 820 | -0.01 (-0.03, 0.01) | 0.36 |
| Model 2 | 820 | -0.01 (-0.02, 0.01) | 0.41 |

Abbreviations: SCFAs; serum short chain fatty acids; GNHS, Guangzhou Nutrition and Health Study.

* β-coefficients were generated from multiple linear regression with the z-score of the relative abundance of *Odoribacter* and natural log-transformed concentrations of SCFAs as exposure and outcome, respectively.

^†^ The model 1 was adjusted for age, gender, body mass index, education, income; and model 2, as model 1 + Bristol scale and total energy intake.

**Table S8. Associations between serum acetic acid and brain structure in the GNHS**

| **Brain structure** | **No. of participants** | **β (95% CI)^*^** | ***P* values** |
| --- | --- | --- | --- |
| Hippocampus_R |  |  |  |
| Model 1^†^ | 185 | 0.14 (0.01, 0.27) | 0.04 |
| Model 2^†^ | 185 | 0.14 (0.01, 0.28) | 0.04 |
| WM |  |  |  |
| Model 1 | 185 | 0.04 (-0.07, 0.14) | 0.49 |
| Model 2 | 185 | 0.04 (-0.06, 0.15) | 0.41 |
| CSF |  |  |  |
| Model 1 | 185 | -0.07 (-0.17, 0.03) | 0.16 |
| Model 2 | 185 | -0.07 (-0.17, 0.02) | 0.14 |

Abbreviations: GNHS, Guangzhou Nutrition and Health Study; WH, white matter; CSF, cerebrospinal fluid; Hippocampus_R, right hippocampus.

* β-coefficients were generated from linear regression with the natural log-transformed concentration of acetic acid and z-scores of volume of different brain regions as exposure and outcome, respectively.

^†^ The model 1 was adjusted for total intracranial volume, age, gender, body mass index, education, income; and model 2, as model 1 + history of stroke.

**Table S9. Distribution of metagenomic features in the GNHS**

| **Code** | **Name** | **Mean relative abundance** | |
| --- | --- | --- | --- |
|  |  | **Normal** | **Mild** |
| s100 | *Bacteroides_dorei* | 3.15248 | 2.48024 |
| s107 | *Bacteroides_massiliensis* | 1.84358 | 1.83662 |
| s110 | *Bacteroides_ovatus* | 2.23855 | 1.53379 |
| s111 | *Bacteroides_plebeius* | 2.17775 | 2.24420 |
| s122 | *Bacteroides_stercoris* | 5.15823 | 4.23962 |
| s124 | *Bacteroides_uniformis* | 5.66702 | 4.45097 |
| s125 | *Bacteroides_vulgatus* | 4.75266 | 3.31000 |
| s156 | *Prevotella_copri* | 5.81730 | 7.21605 |
| s168 | *Alistipes_putredinis* | 4.28771 | 3.79061 |
| s335 | *Eubacterium_rectale* | 2.69155 | 5.30464 |
| s397 | *Faecalibacterium_prausnitzii* | 5.35493 | 5.59088 |
| s400 | *Ruminococcus_bromii* | 2.14515 | 2.33738 |
| s408 | *Subdoligranulum_unclassified* | 3.83473 | 3.60417 |
| s444 | *Megamonas_unclassified* | 1.92030 | 2.36984 |
| s575 | *Escherichia_coli* | 4.69467 | 5.20179 |
| s578 | *Escherichia_unclassified* | 1.90752 | 1.77258 |
| s581 | *Klebsiella_pneumoniae* | 1.77938 | 1.09813 |
| s98 | *Bacteroides_coprocola* | 1.78431 | 1.25361 |
| ANAGLYCOLYSISPWY | glycolysis III (from glucose) | 0.00035 | 0.00034 |
| ARGSYNPWY | L-arginine biosynthesis I (via L-ornithine) | 0.00025 | 0.00025 |
| AROPWY | chorismate biosynthesis I | 0.00047 | 0.00050 |
| BRANCHEDCHAINAASYNPWY | superpathway of branched amino acid biosynthesis | 0.00031 | 0.00033 |
| CALVINPWY | Calvin-Benson-Bassham cycle | 0.00034 | 0.00036 |
| CMET2PWY | N10-formyl-tetrahydrofolate biosynthesis | 0.00038 | 0.00036 |
| COAPWY1 | coenzyme A biosynthesis II (mammalian) | 0.00056 | 0.00056 |
| COMPLETEAROPWY | superpathway of aromatic amino acid biosynthesis | 0.00047 | 0.00050 |
| DTDPRHAMSYNPWY | dTDP-L-rhamnose biosynthesis I | 0.00029 | 0.00030 |
| ILEUSYNPWY | L-isoleucine biosynthesis I (from threonine) | 0.00061 | 0.00061 |
| NONMEVIPPPWY | methylerythritol phosphate pathway I | 0.00057 | 0.00058 |
| NONOXIPENTPWY | pentose phosphate pathway (non-oxidative branch) | 0.00027 | 0.00029 |

**Table S9 (continued)**

| **Code** | **Name** | **Mean relative abundance** | |
| --- | --- | --- | --- |
|  |  | **Normal** | **Mild** |
| PANTOPWY | phosphopantothenate biosynthesis I | 0.00052 | 0.00050 |
| PANTOSYNPWY | pantothenate and coenzyme A biosynthesis I | 0.00024 | 0.00023 |
| PEPTIDOGLYCANSYNPWY | peptidoglycan biosynthesis I (meso-diaminopimelate containing) | 0.00063 | 0.00063 |
| PWY01296 | purine ribonucleosides degradation | 0.00032 | 0.00035 |
| PWY01319 | CDP-diacylglycerol biosynthesis II | 0.00049 | 0.00050 |
| PWY0162 | superpathway of pyrimidine ribonucleotides de novo biosynthesis | 0.00022 | 0.00021 |
| PWY0166 | superpathway of pyrimidine deoxyribonucleotides de novo biosynthesis (E. coli) | 0.00023 | 0.00022 |
| PWY0845 | superpathway of pyridoxal 5'-phosphate biosynthesis and salvage | 0.00023 | 0.00021 |
| PWY1042 | glycolysis IV (plant cytosol) | 0.00043 | 0.00044 |
| PWY2942 | L-lysine biosynthesis III | 0.00055 | 0.00056 |
| PWY3001 | superpathway of L-isoleucine biosynthesis I | 0.00027 | 0.00027 |
| PWY3841 | folate transformations II | 0.00040 | 0.00038 |
| PWY5097 | L-lysine biosynthesis VI | 0.00059 | 0.00059 |
| PWY5103 | L-isoleucine biosynthesis III | 0.00027 | 0.00029 |
| PWY5667 | CDP-diacylglycerol biosynthesis I | 0.00049 | 0.00050 |
| PWY5686 | UMP biosynthesis | 0.00068 | 0.00070 |
| PWY5695 | urate biosynthesis/inosine 5'-phosphate degradation | 0.00050 | 0.00047 |
| PWY5973 | cis-vaccenate biosynthesis | 0.00030 | 0.00030 |
| PWY6121 | 5-aminoimidazole ribonucleotide biosynthesis I | 0.00046 | 0.00047 |
| PWY6122 | 5-aminoimidazole ribonucleotide biosynthesis II | 0.00044 | 0.00046 |
| PWY6123 | inosine-5'-phosphate biosynthesis I | 0.00024 | 0.00023 |
| PWY6125 | superpathway of guanosine nucleotides de novo biosynthesis II | 0.00025 | 0.00024 |
| PWY6126 | superpathway of adenosine nucleotides de novo biosynthesis II | 0.00032 | 0.00031 |
| PWY6151 | S-adenosyl-L-methionine cycle I | 0.00057 | 0.00059 |
| PWY6163 | chorismate biosynthesis from 3-dehydroquinate | 0.00048 | 0.00052 |

**Table S9 (continued)**

| **Code** | **Name** | **Mean relative abundance** | | |
| --- | --- | --- | --- | --- |
|  |  | **Normal** | | **Mild** |
| PWY6277 | superpathway of 5-aminoimidazole ribonucleotide biosynthesis | 0.00044 | 0.00046 | |
| PWY6385 | peptidoglycan biosynthesis III (mycobacteria) | 0.00041 | 0.00039 | |
| PWY6386 | UDP-N-acetylmuramoyl-pentapeptide biosynthesis II (lysine-containing) | 0.00066 | 0.00066 | |
| PWY6387 | UDP-N-acetylmuramoyl-pentapeptide biosynthesis I (meso-diaminopimelate containing) | 0.00063 | 0.00064 | |
| PWY6609 | adenine and adenosine salvage III | 0.00021 | 0.00021 | |
| PWY6700 | queuosine biosynthesis | 0.00056 | 0.00055 | |
| PWY6703 | preQ0 biosynthesis | 0.00039 | 0.00036 | |
| PWY6737 | starch degradation V | 0.00050 | 0.00054 | |
| PWY6897 | thiamin salvage II | 0.00026 | 0.00025 | |
| PWY7111 | pyruvate fermentation to isobutanol (engineered) | 0.00061 | 0.00062 | |
| PWY7208 | superpathway of pyrimidine nucleobases salvage | 0.00025 | 0.00024 | |
| PWY7219 | adenosine ribonucleotides de novo biosynthesis | 0.00086 | 0.00090 | |
| PWY7221 | guanosine ribonucleotides de novo biosynthesis | 0.00065 | 0.00066 | |
| PWY7228 | superpathway of guanosine nucleotides de novo biosynthesis I | 0.00030 | 0.00028 | |
| PWY7229 | superpathway of adenosine nucleotides de novo biosynthesis I | 0.00038 | 0.00037 | |
| PWY724 | superpathway of L-lysine, L-threonine and L-methionine biosynthesis II | 0.00038 | 0.00038 | |
| PWY7357 | thiamin formation from pyrithiamine and oxythiamine (yeast) | 0.00027 | 0.00028 | |
| PWY7400 | L-arginine biosynthesis IV (archaebacteria) | 0.00025 | 0.00026 | |
| PWY7663 | gondoate biosynthesis (anaerobic) | 0.00028 | 0.00027 | |
| PWY841 | superpathway of purine nucleotides de novo biosynthesis I | 0.00021 | 0.00021 | |
| THRESYNPWY | superpathway of L-threonine biosynthesis | 0.00026 | 0.00025 | |
| TRNACHARGINGPWY | tRNA charging | 0.00034 | 0.00035 | |
| VALSYNPWY | L-valine biosynthesis | 0.00061 | 0.00061 | |

Abbreviations: GNHS, Guangzhou Nutrition and Health Study.

**Table S10. Associations between intra-individual alterations in gut microbial composition and cognitive impairment in the GNHS**

| **Brain structure** | **No. of participants** | **OR (95% CI)^*^** | ***P* values** |
| --- | --- | --- | --- |
| Questionable *vs.* Normal |  |  |  |
| Model 1^†^ | 272 | 1.26 (0.90, 1.76) | 0.172 |
| Model 2^†^ | 272 | 1.26 (0.89, 1.77) | 0.193 |
| Mild *vs.* Normal |  |  |  |
| Model 1 | 272 | 1.94 (1.23, 3.06) | 0.005 |
| Model 2 | 272 | 1.89 (1.18, 3.03) | 0.008 |

Abbreviations: GNHS, Guangzhou Nutrition and Health Study; OR, odds ratio; CI, confidence interval.

^*^ ORs were generated from multinomial logistic regression with the normal group as reference. The Bray–Curtis dissimilarities between paired samples within individuals were standardized as z-score before the regression analysis.

^†^ The covariates in the model 1 included age, gender, body mass index, education, and income; and we further included Bristol scale and history of stroke as covariates in the model 2.

**Table S11. Metagenomic and metabolomic features associated with cognitive impairment in the GNHS**

| **Models^*^** | **β coefficients** |
| --- | --- |
| **Species only** |  |
| *Eubacterium rectale* | 0.078 |
| *Ruminococcuobeum* | 0.024 |
| *Dorea longicatena* | 0.021 |
| *Coprobacilluunclassified* | 0.013 |
| *Citrobacter unclassified* | -0.044 |
| **Pathways only** |  |
| Glycogen biosynthesis I (from ADP-D-Glucose) | 0.056 |
| Petroselinate biosynthesis | 0.052 |
| Formaldehyde oxidation I | 0.061 |
| **Serum metabolites only** |  |
| L-Phenylalanine | -0.153 |
| Glyceric acid | -0.028 |
| Methylglutaric acid | 0.138 |
| Hyodeoxycholic acid | 0.016 |
| **Combined model** |  |
| L-Phenylalanine | -0.279 |
| Glyceric acid | -0.036 |
| Methylglutaric acid | 0.254 |
| Hyodeoxycholic acid | 0.107 |
| *Dorea longicatena* | 0.046 |
| Glycogen biosynthesis I (from ADP-D-Glucose) | 0.167 |
| Petroselinate biosynthesis | 0.021 |
| Formaldehyde oxidation I | 0.037 |

Abbreviations: GNHS, Guangzhou Nutrition and Health Study.

**^*^** All features were standardized as z-scores for the least absolute shrinkage and selection operator (LASSO) models.

**Table S12. Correlations of metagenomic and metabolic features with MMSE domains in the GNHS**

| **Metagenomic and metabolic features** | **MMSE domains** | **Rho** | ***P* values** | **FDR** |
| --- | --- | --- | --- | --- |
| *Dorea longicatena* | Orientation | -0.067 | 0.058 | 0.192 |
| *Dorea longicatena* | Registration | -0.037 | 0.298 | 0.496 |
| *Dorea longicatena* | Attention and calculation | 0.019 | 0.588 | 0.789 |
| *Dorea longicatena* | Delayed recall | -0.016 | 0.658 | 0.809 |
| *Dorea longicatena* | Language | -0.097 | 0.006 | 0.049 |
| Glycogen biosynthesis I (from ADP-D-Glucose) | Orientation | -0.012 | 0.736 | 0.842 |
| Glycogen biosynthesis I (from ADP-D-Glucose) | Registration | -0.051 | 0.150 | 0.316 |
| Glycogen biosynthesis I (from ADP-D-Glucose) | Attention and calculation | -0.054 | 0.126 | 0.280 |
| Glycogen biosynthesis I (from ADP-D-Glucose) | Delayed recall | -0.069 | 0.051 | 0.184 |
| Glycogen biosynthesis I (from ADP-D-Glucose) | Language | -0.076 | 0.031 | 0.136 |
| Petroselinate biosynthesis | Orientation | -0.006 | 0.862 | 0.957 |
| Petroselinate biosynthesis | Registration | -0.016 | 0.656 | 0.809 |
| Petroselinate biosynthesis | Attention and calculation | -0.028 | 0.431 | 0.639 |
| Petroselinate biosynthesis | Delayed recall | -0.054 | 0.125 | 0.280 |
| Petroselinate biosynthesis | Language | -0.092 | 0.009 | 0.052 |
| Formaldehyde oxidation I | Orientation | -0.032 | 0.367 | 0.565 |
| Formaldehyde oxidation I | Registration | -0.059 | 0.092 | 0.246 |
| Formaldehyde oxidation I | Attention and calculation | -0.003 | 0.933 | 0.957 |
| Formaldehyde oxidation I | Delayed recall | -0.064 | 0.068 | 0.197 |
| Formaldehyde oxidation I | Language | -0.141 | 5.47E-05 | 0.0010943 |
| L_Phenylalanine | Orientation | -0.004 | 0.915 | 0.957 |
| L_Phenylalanine | Registration | 0.000 | 0.994 | 0.994 |
| L_Phenylalanine | Attention and calculation | 0.045 | 0.202 | 0.385 |
| L_Phenylalanine | Delayed recall | 0.082 | 0.019 | 0.096 |
| L_Phenylalanine | Language | 0.112 | 0.001 | 0.019 |
| Glyceric acid | Orientation | -0.057 | 0.107 | 0.269 |
| Glyceric acid | Registration | 0.043 | 0.224 | 0.390 |
| Glyceric acid | Attention and calculation | -0.012 | 0.731 | 0.842 |
| Glyceric acid | Delayed recall | 0.032 | 0.357 | 0.565 |
| Glyceric acid | Language | 0.281 | 4.39E-16 | 1.76E-14 |

**Table S12 (continued)**

| **Metagenomic and metabolic features** | **MMSE domains** | **Rho** | ***P* values** | **FDR** |
| --- | --- | --- | --- | --- |
| Methylglutaric acid | Orientation | 0.0037 | 0.9159 | 0.9572 |
| Methylglutaric acid | Registration | -0.0429 | 0.2234 | 0.3902 |
| Methylglutaric acid | Attention and calculation | -0.0454 | 0.1971 | 0.3854 |
| Methylglutaric acid | Recalling | -0.0641 | 0.0688 | 0.1966 |
| Methylglutaric acid | Language | -0.0960 | 0.0063 | 0.0492 |
| Hyodeoxycholic acid | Orientation | -0.0189 | 0.5916 | 0.7888 |
| Hyodeoxycholic acid | Registration | -0.0259 | 0.4614 | 0.6591 |
| Hyodeoxycholic acid | Attention and calculation | -0.0691 | 0.0498 | 0.1840 |
| Hyodeoxycholic acid | Recalling | -0.0942 | 0.0074 | 0.0492 |
| Hyodeoxycholic acid | Language | -0.0152 | 0.6672 | 0.8087 |

Abbreviations: MMSE, the Mini-Mental State Examination; GNHS, Guangzhou Nutrition and Health Study; FDR, fase discovery rate.

Semi-partial correlation was used to estimate the correlations of the LASSO-identified metagenomic and metabolic features with MMSE domains, adjusted for age, gender, body mass index, education, and income. FDR using Benjamini–Hochberg method was calculated to correct the multiple testing.

**Table S13. Correlations between metagenomic features and metabolites in the GNHS**

| **Metagenomic features** | **Metabolites** | **Rho** | ***P* values** | **FDR** |
| --- | --- | --- | --- | --- |
| *Dorea longicatena* | L-Phenylalanine | -0.095 | 0.007 | 0.020 |
| *Dorea longicatena* | Glyceric acid | -0.071 | 0.042 | 0.085 |
| *Dorea longicatena* | Methylglutaric acid | 0.023 | 0.520 | 0.694 |
| *Dorea longicatena* | Hyodeoxycholic acid | 0.1615 | 3.94E-06 | 2.52E-05 |
| Glycogen biosynthesis I (from ADP-D-Glucose) | L-Phenylalanine | 0.007 | 0.841 | 0.841 |
| Glycogen biosynthesis I (from ADP-D-Glucose) | Glyceric acid | -0.093 | 0.008 | 0.020 |
| Glycogen biosynthesis I (from ADP-D-Glucose) | Methylglutaric acid | 0.013 | 0.703 | 0.750 |
| Glycogen biosynthesis I (from ADP-D-Glucose) | Hyodeoxycholic acid | 0.104 | 0.003 | 0.011 |
| Petroselinate biosynthesis | L-Phenylalanine | -0.066 | 0.060 | 0.100 |
| Petroselinate biosynthesis | Glyceric acid | -0.025 | 0.479 | 0.667 |
| Petroselinate biosynthesis | Methylglutaric acid | 0.014 | 0.701 | 0.750 |
| Petroselinate biosynthesis | Hyodeoxycholic acid | 0.2151 | 6.54E-10 | 1.32E-08 |
| Formaldehyde oxidation I | L-Phenylalanine | -0.071 | 0.043 | 0.085 |
| Formaldehyde oxidation I | Glyceric acid | -0.129 | 0.000 | 0.001 |
| Formaldehyde oxidation I | Methylglutaric acid | 0.025 | 0.479 | 0.667 |
| Formaldehyde oxidation I | Hyodeoxycholic acid | 0.1467 | 2.85E-05 | 0.00015189 |

Abbreviations: GNHS, Guangzhou Nutrition and Health Study; FDR, fase discovery rate.

Semi-partial correlation was used to estimate the correlations between the LASSO-identified metagenomic features and LASSO-identified metabolites, adjusted for age, gender, body mass index, education, and income. FDR using Benjamini–Hochberg method was calculated to correct the multiple testing.

**Supplementary Figure Legends**

**
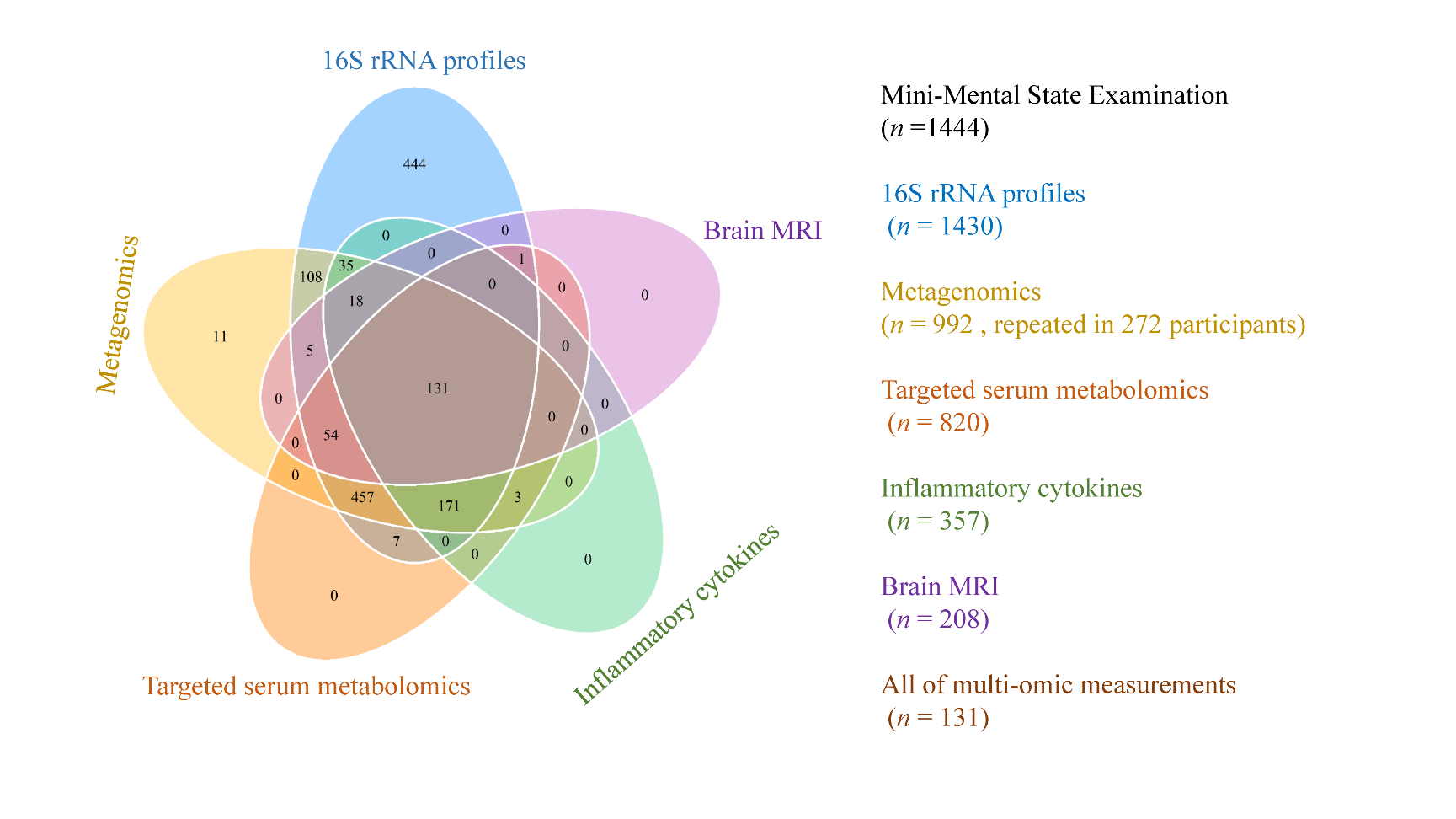
**

**Figure S1. Overview of the multi-omics datasets of the GNHS.** The Venn plot shows overlaps between the multi-omics datasets. The numbers in each circle represent the corresponding number of the individuals. The annotation beside the circles presents the total samples of each dataset. The blue, yellow, orange, green, and purple circles represent the datasets of 16S rRNA gene sequencing, metagenomics, targeted serum metabolomics, inflammatory cytokines, and brain magnetic resonance imaging (MRI), respectively. All the participants (*n* = 1444) had attended cognitive screening based on the Mini-Mental State Examination. Abbreviation: GNHS, Guangzhou Nutrition and Health Study.

**
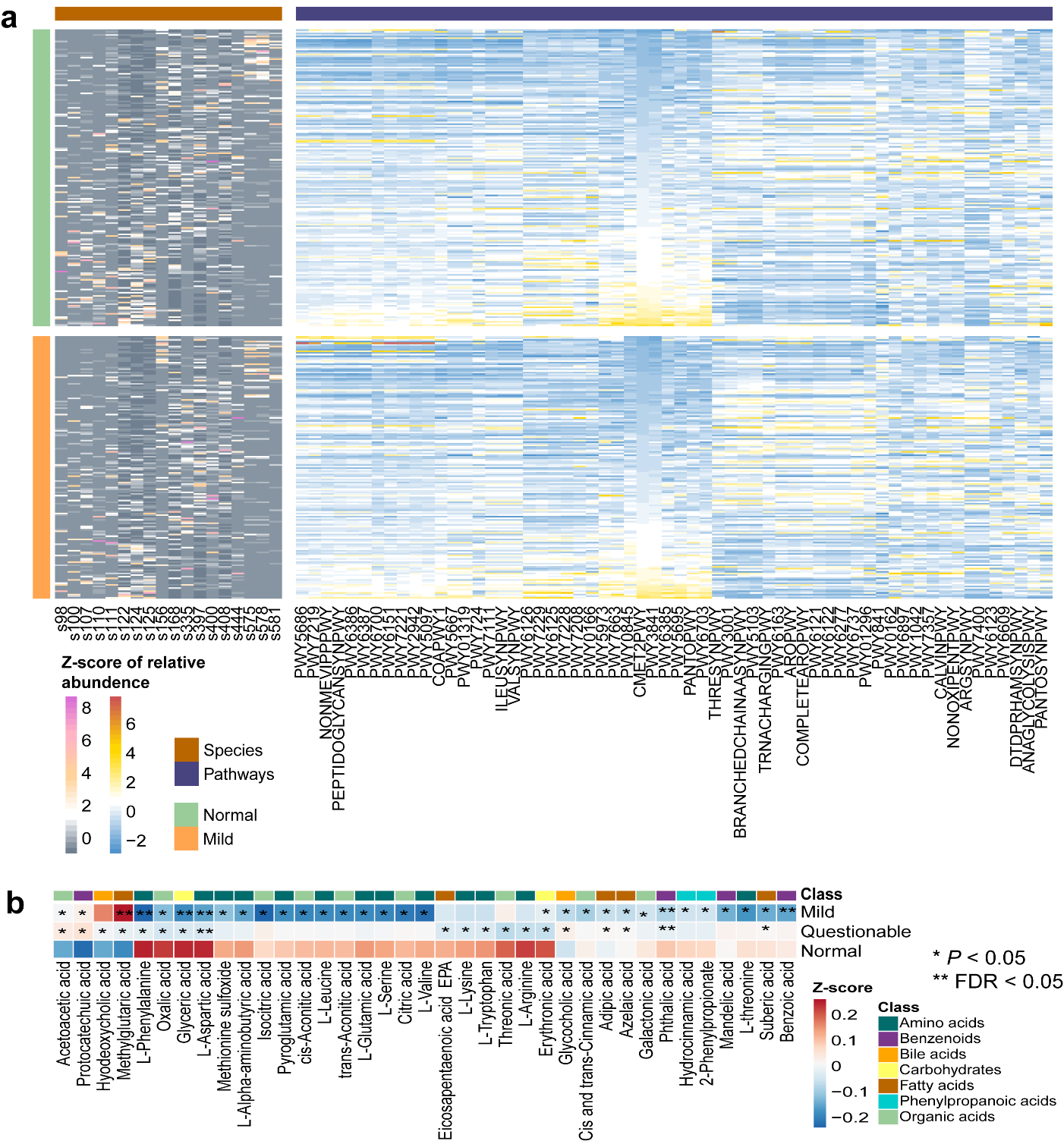
**

**Figure S2. Distribution of metagenomic and metabolomic features in the GNHS. (a)** We sorted species (or pathways) according to their relative abundance from highest to lowest, and selected those with a sum of relative abundance accounted for 60% of all species (or pathways). As a result, 18 species and 60 pathways were selected to show in the heatmap (Table S9). The intensity of the colors represents z-scores of relative abundances. Each row of the heatmap represents a sample. **(b)** Serum metabolites alter in participants with cognitive impairment. *P* values were generated from Kruskal–Wallis test. The heatmap shows significant difference(s) in at least one of the questionable and mild groups compared with the normal group. Intensity of the colors represents the average z-scores of metabolite concentrations in each group. False discovery rate (FDR) were calculated using Benjamini–Hochberg method with a target rate of 0.05. Abbreviation: GNHS, Guangzhou Nutrition and Health Study**.**


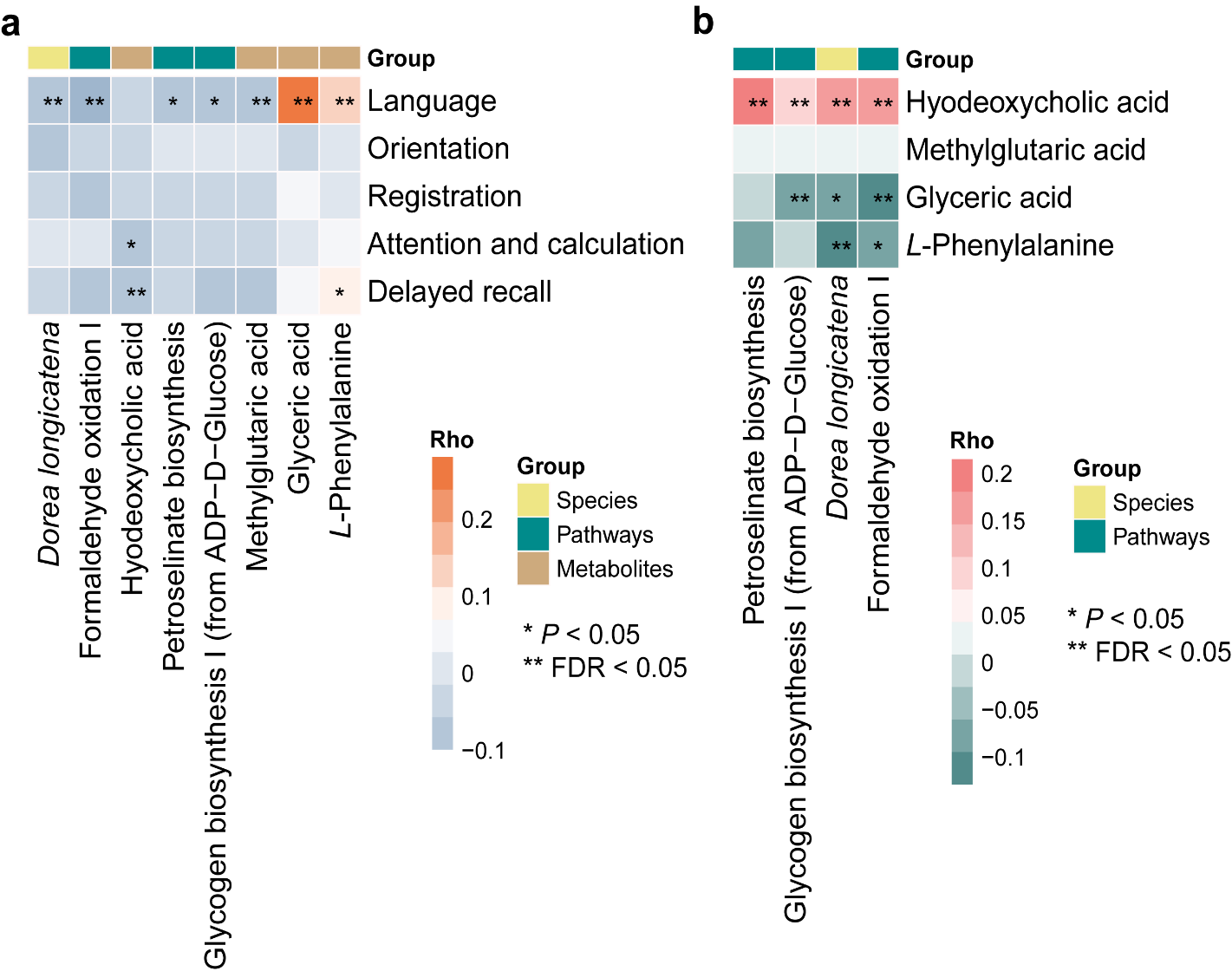


**Figure S3. Correlation analyses on cognition-related metagenomic and metabolic traits in the GNHS.** The heatmaps show the result of semi-partial correlation of: **(a)** metagenomic and metabolic features selected from the combined LASSO mode l with MMSE domains, and **(b)** aforementioned metagenomic features with aforementioned metabolites. False discovery rate (FDR) was calculated using Benjamini–Hochberg method. Abbreviation: GNHS, Guangzhou Nutrition and Health Study; MMSE, Mini-Mental State Examination**.**

**
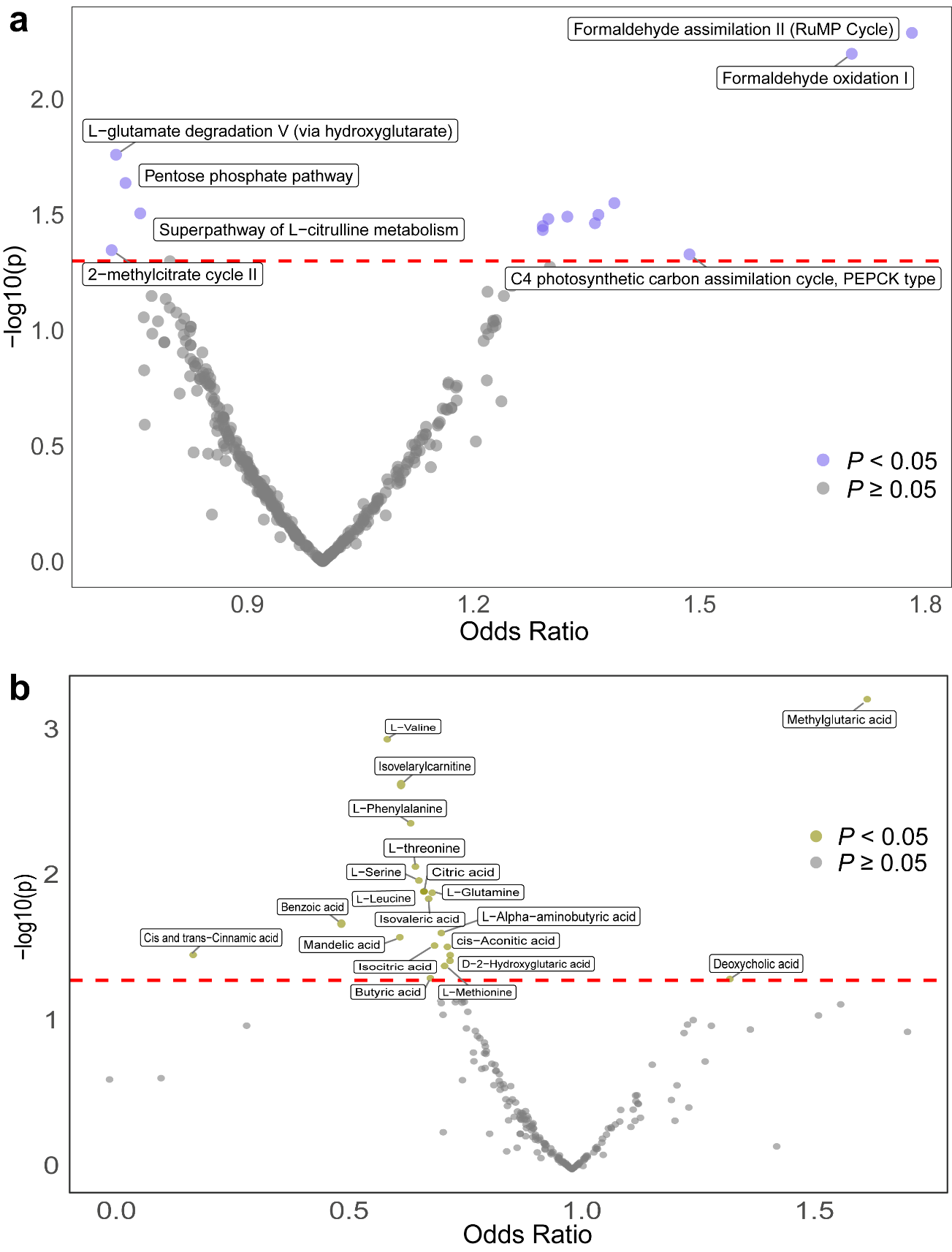
**

**Figure S4. Association of metagenomic pathways and serum metabolomics with cognitive function in the GNHS.** The volcano plot shows metagenomic pathways **(a)** and serum metabolites **(b)** associated with cognitive impairment. The logistic regression was adjusted for age, gender, body mass index, education, income, history of stroke, and time lag between sample collection and cognitive assessment. The x axis shows odds ratios and the y axis indicates -log (base 10) of *p* values. Abbreviation: GNHS, Guangzhou Nutrition and Health Study**.**


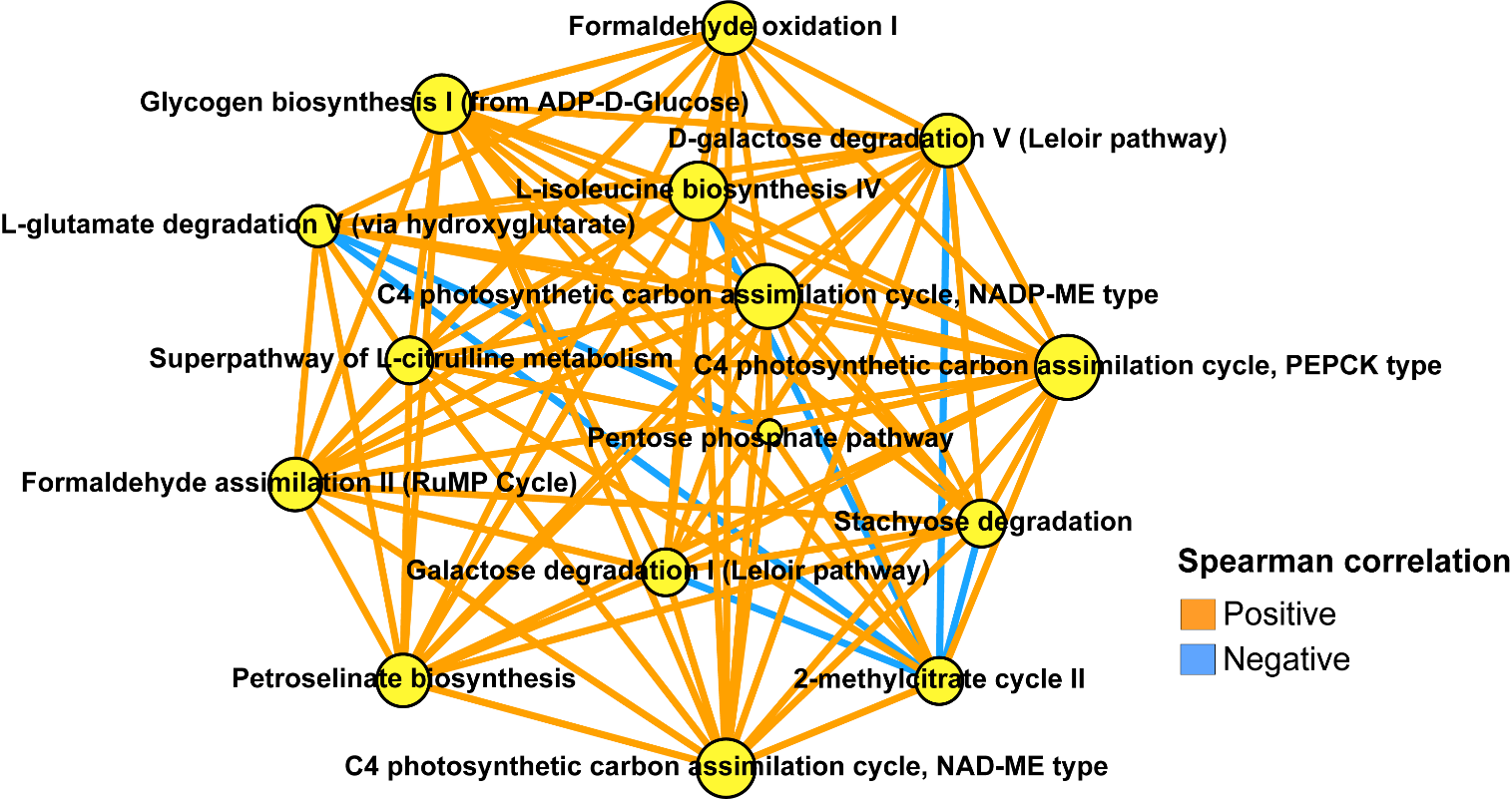


**Figure S5. Networks of metagenomic pathways in the GNHS.** The network showing significant (FDR < 0.05) Spearman correlations among the metagenomic pathways. Spearman correlation analyses were conducted among 992 participants. Sizes of nodes represent the number of connections between the corresponding pathway with others. Orange edge, Spearman correlation coefficient > 0; blue edge, Spearman correlation coefficient < 0. Abbreviations: GNHS, Guangzhou Nutrition and Health Study.

**References**

1. Katzman R, Zhang MY, Ouang Ya Q, Wang ZY, Liu WT, Yu E, et al. A Chinese version of the Mini-Mental State Examination; impact of illiteracy in a Shanghai dementia survey. J Clin Epidemiol. 1988;41(10):971-8.

2. Li B, He Y, Ma J, Huang P, Du J, Cao L, et al. Mild cognitive impairment has similar alterations as Alzheimer's disease in gut microbiota. Alzheimers Dement. 2019;15(10):1357-66.

3. Popkin BM, Du S, Zhai F, Zhang B. Cohort Profile: The China Health and Nutrition Survey--monitoring and understanding socio-economic and health change in China, 1989-2011. Int J Epidemiol. 2010;39(6):1435-40.

4. Brandt J, Welsh KA, Breitner JC, Folstein MF, Helms M, Christian JC. Hereditary influences on cognitive functioning in older men. A study of 4000 twin pairs. Arch Neurol. 1993;50(6):599-603.

5. Callahan BJ, McMurdie PJ, Rosen MJ, Han AW, Johnson AJ, Holmes SP. DADA2: High-resolution sample inference from Illumina amplicon data. Nat Methods. 2016;13(7):581-3.

6. Truong DT, Franzosa EA, Tickle TL, Scholz M, Weingart G, Pasolli E, et al. MetaPhlAn2 for enhanced metagenomic taxonomic profiling. Nat Methods. 2015;12(10):902-3.

7. Caspi R, Billington R, Ferrer L, Foerster H, Fulcher CA, Keseler IM, et al. The MetaCyc database of metabolic pathways and enzymes and the BioCyc collection of pathway/genome databases. Nucleic Acids Res. 2016;44(D1):D471-80.

8. Shuai M, Zhang G, Zeng FF, Fu Y, Liang X, Yuan L, et al. Human Gut Antibiotic Resistome and Progression of Diabetes. Adv Sci (Weinh). 2022;9(11):e2104965.

9. Yu Q, McCall DM, Homayouni R, Tang L, Chen Z, Schoff D, et al. Age-associated increase in mnemonic strategy use is linked to prefrontal cortex development. Neuroimage. 2018;181:162-9.

10. Pirlich M, Hofer C, Weise CM, Stockert A, Thone-Otto A, Garthe A, et al. Hippocampal gray matter volume in the long-term course after transient global amnesia. Neuroimage Clin. 2021;30:102586.

11. Tzourio-Mazoyer N, Landeau B, Papathanassiou D, Crivello F, Etard O, Delcroix N, et al. Automated anatomical labeling of activations in SPM using a macroscopic anatomical parcellation of the MNI MRI single-subject brain. Neuroimage. 2002;15(1):273-89.
